# Supplementary material for: Metabolite annotation from knowns to unknowns through knowledge-guided multi-layer metabolic networking
Source: Nat Commun. 2022 Nov 4;13:6656. doi: 10.1038/s41467-022-34537-6 (PMC9636193; doi:10.1038/s41467-022-34537-6)
Supplement: Supplementary file 1 — Supplementary Information [file 41467_2022_34537_MOESM1_ESM.pdf]

Supplementary information for

Metabolite annotation from knowns to unknowns through knowledge-guided multi-layer metabolic networking

Zhiwei Zhou[1,†], Mingdu Luo[1,2,†], Haosong Zhang[1,2], Yandong Yin[1], Yuping Cai[1], and Zheng-Jiang Zhu[1,3,]*

[1] Interdisciplinary Research Center on Biology and Chemistry, Shanghai Institute of Organic Chemistry, Chinese Academy of Sciences, Shanghai, 200032 China

[2] University of Chinese Academy of Sciences, Beijing, 100049 China

[3] Shanghai Key Laboratory of Aging Studies, Shanghai, 201210 China

[†] These authors contributed equally

Corresponding Author

Correspondence should be addressed to Z.J.Z (jiangzhu@sioc.ac.cn)

List for Supplementary Figures

Supplementary Figure 1. Curation of knowledge-based metabolic reaction network (KMRN) with *in-silico* enzymatic reactions.

Supplementary Figure 2. Statistics of linked nodes in MS/MS similarity network or knowledge-guided MS/MS similarity network.

Supplementary Figure 3. The construction and optimization of global peak annotation network.

Supplementary Figure 4. Flowchart for the optimization and filtering of subnetworks in the global peak correlation network.

Supplementary Figure 5. The workflow of accuracy evaluation with a manually curated data set.

Supplementary Figure 6. Comparison between MetDNA1 and KGMN in different biological samples, including NIST human urine, NIST human plasma, BV2 cells, head tissues of fruit fly, and 200STD spiked mouse liver tissues.

Supplementary Figure 7. Benchmark comparison between CAMERA and KGMN for annotating ion forms of metabolic peaks.

Supplementary Figure 8. KGMN recognized the in-source fragments of N4-Acetylcytidine.

Supplementary Figure 9. Examples of different ion form recognition and peak assignment in KGMN.

Supplementary Figure 10. Knowledge-guided multi-layer networks of 46std_mix data sets.

Supplementary Figure 11. Validation examples of annotated unknowns in 46std_mix data sets.

Supplementary Figure 12. Knowledge-guided MS/MS similarity network of NIST human urine sample.

Supplementary Figure 13. Global peak correlation network of NIST human urine sample.

Supplementary Figure 14. Global annotation of unknown metabolites in negative mode and validation examples of unknowns using *in-silico* MS/MS tools.

Supplementary Figure 15. The repository-mining and structural validations of 3 recurrent unknown metabolites.

Supplementary Figure 16. Curated unknown metabolites and reaction pairs in the knowledge-based metabolic reaction network (KMRN).

List for Supplementary Table

Supplementary Table 1. The supported data processing tools with KGMN

Supplementary Table 2. Statistics of global peak annotation optimization to improve annotation accuracy.

Supplementary Table 3. Statistics of biotransformation types in 46std_mix data set.

Supplementary Table 4. Statistics of annotated peaks in different biological samples.

Supplementary Table 5. Statistics of unknown biotransformation types in NIST urine data set.

List for Supplementary Notes

Supplementary Note 1: Tutorial of KGMN result visualization and analysis.

Supplementary Note 2: Tutorial of validating KGMN unknowns with repository mining.

Supplementary Note 3: Tutorial of integrating KGMN results with other in-silico MS/MS workflows.

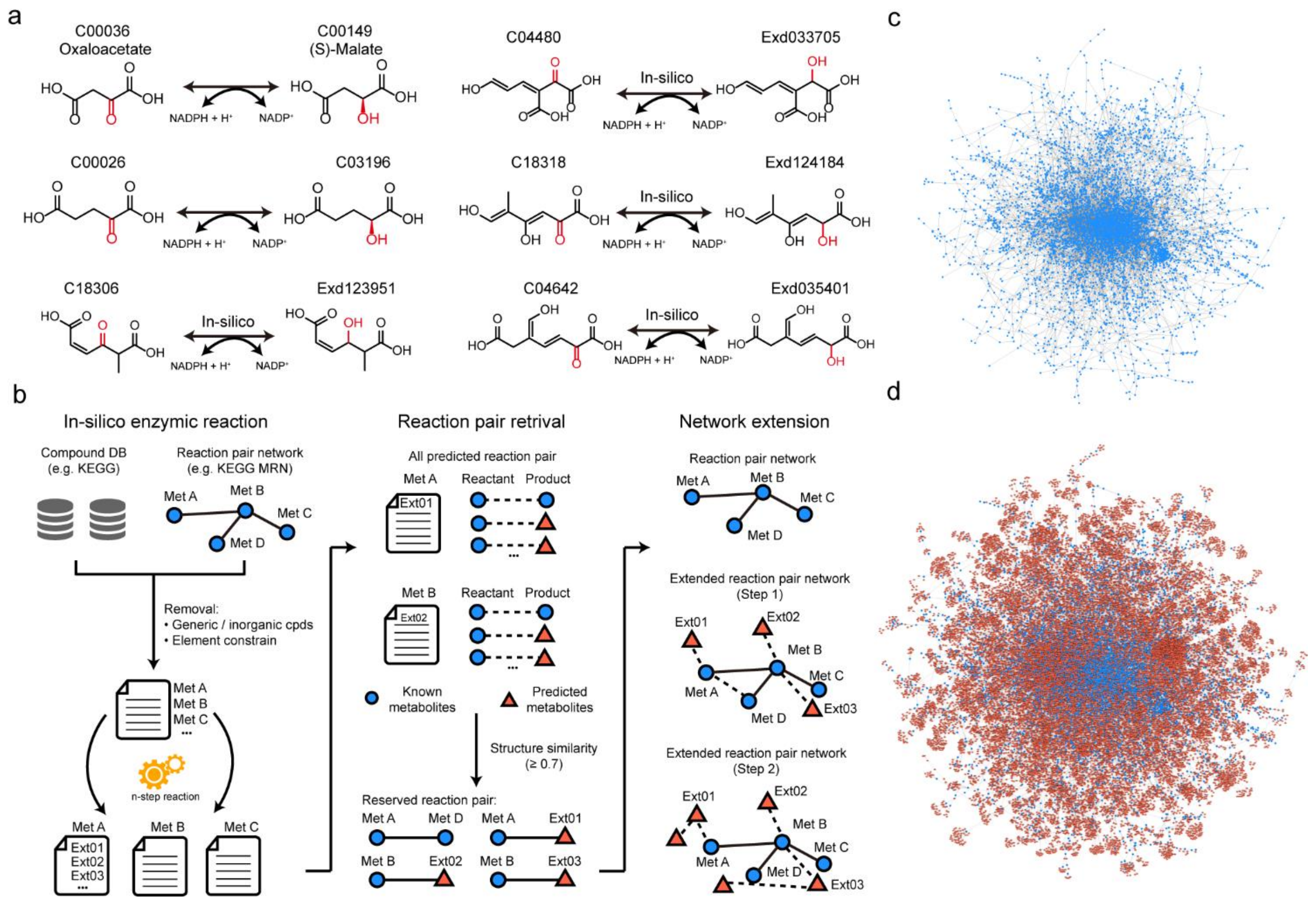

Supplementary Figure 1. Curation of knowledge-based metabolic reaction network (KMRN) with *in-silico* enzymatic reactions. (**a**) Examples for the curation of unknown metabolites through *in-silico* enzymatic reaction; (**b**) The workflow to curate the knowledge-based metabolic reaction network with *in-silico* enzymatic reactions. The known metabolites and reaction pairs were downloaded from the KEGG database, while the unknown metabolites were curated through *in-silico* enzymatic reactions. The reactant and product were paired and filtered with structural similarity. The knowledge-based metabolic reaction network was linked to the known metabolic reaction network. (**c-d**) Knowledge-based metabolic reaction networks: (**c**) known metabolites are connected through known reactions (6,397 nodes and 8,129 edges); (**d**) known and unknown metabolites are connected with known or *in-silico* reactions (41,336 nodes and 52,137 edges). The largest subnetwork is shown here.

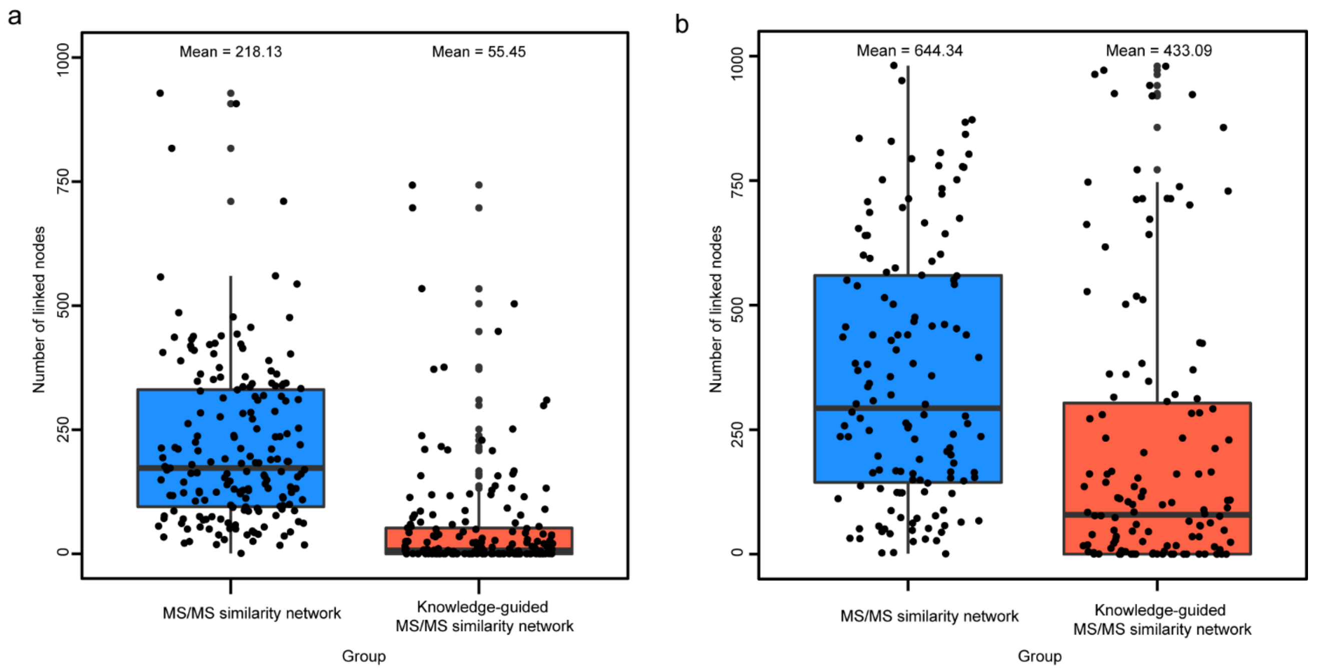

Supplementary Figure 2. Statistics of linked nodes in MS/MS similarity network or knowledge-guided MS/MS similarity network in positive (**a**) and negative modes (**b**), respectively. The linked nodes from seed metabolites in NIST human urine sample (N=181 and 163 in positive and negative modes, respectively) were included here. The cutoff of MS/MS similarity score is defined as 0.5. Neighbor metabolites within 3 steps were considered in knowledge-guided MS/MS similarity network. The lower, middle and upper lines in box plots (**a, b**) correspond to 25th, 50th and 75th quartiles, and the whiskers extend to the most extreme data point within 1.5 interquartile range (IQR).

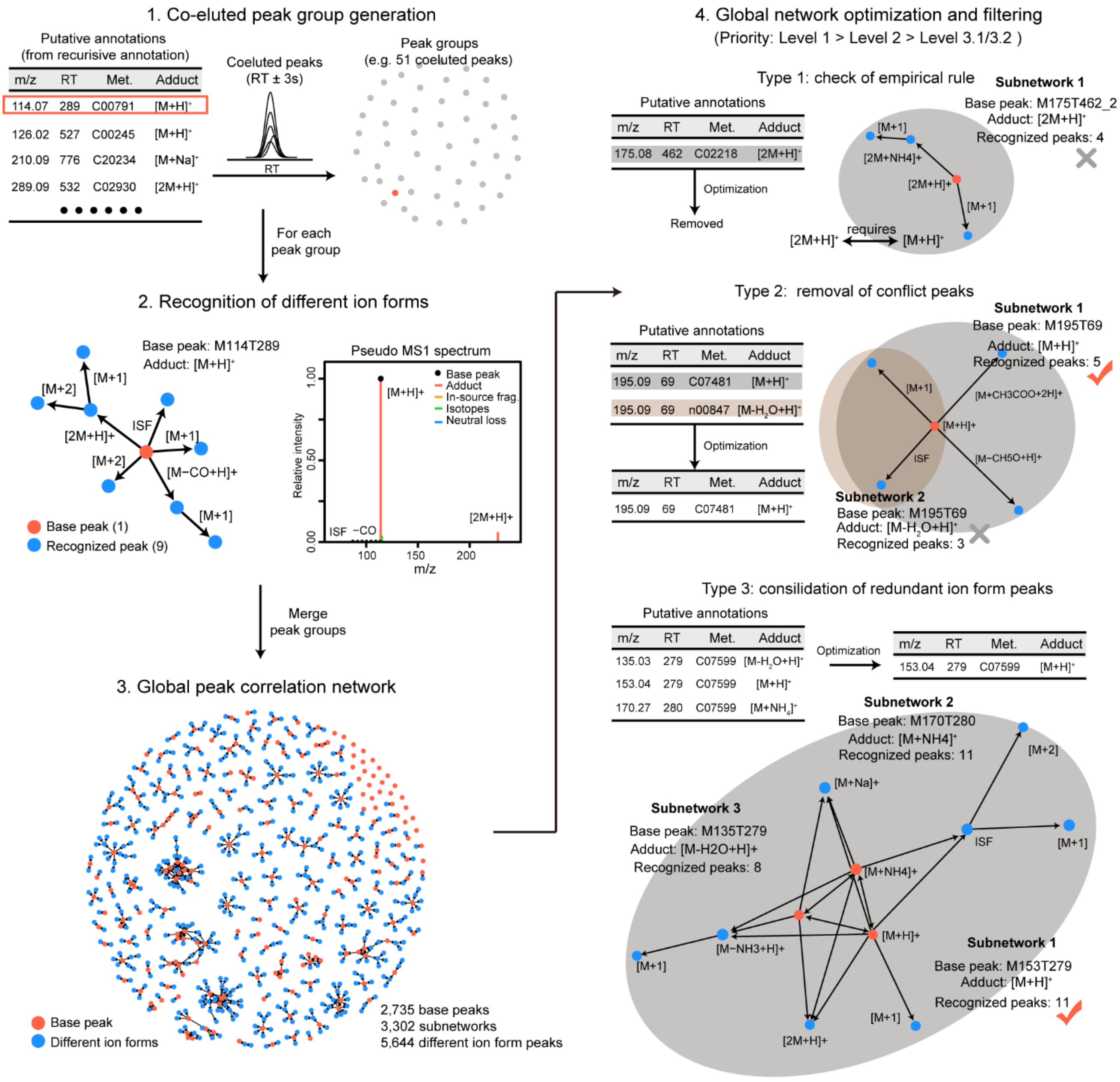

Supplementary Figure 3. The construction and optimization of global peak annotation network. **Step 1**: co-eluted peaks are extracted as one peak group according to the putative metabolite annotations in knowledge-guided MS/MS similarity network; **Step 2**: recognition of different ion forms to build the subnetwork, including adducts, isotopes, in-source fragments and neutral losses; **Step 3**: all recognized subnetworks are merged as a global peak correlation network; **Step 4**: global optimization and conflict resolving to improve the peak annotation accuracy. Three types of conflict annotations are checked and resolved, including empirical rule, removal of conflict peaks and annotations, and consolidation of redundant ion form peaks.

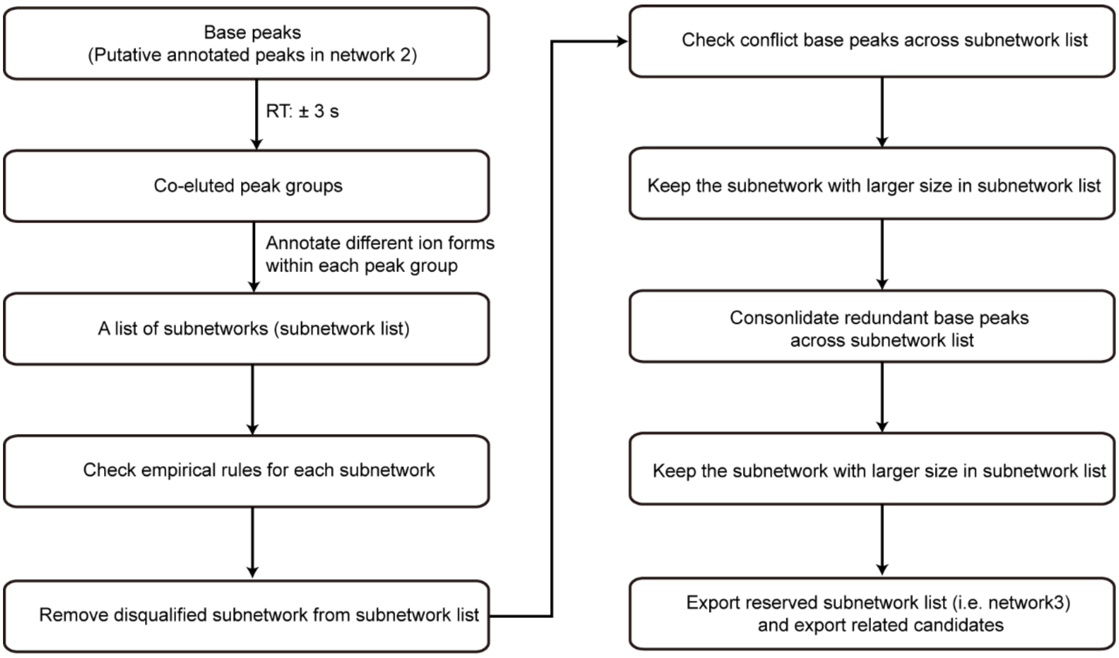

Supplementary Figure 4. Flowchart for the optimization and filtering of subnetworks in the global peak correlation network.

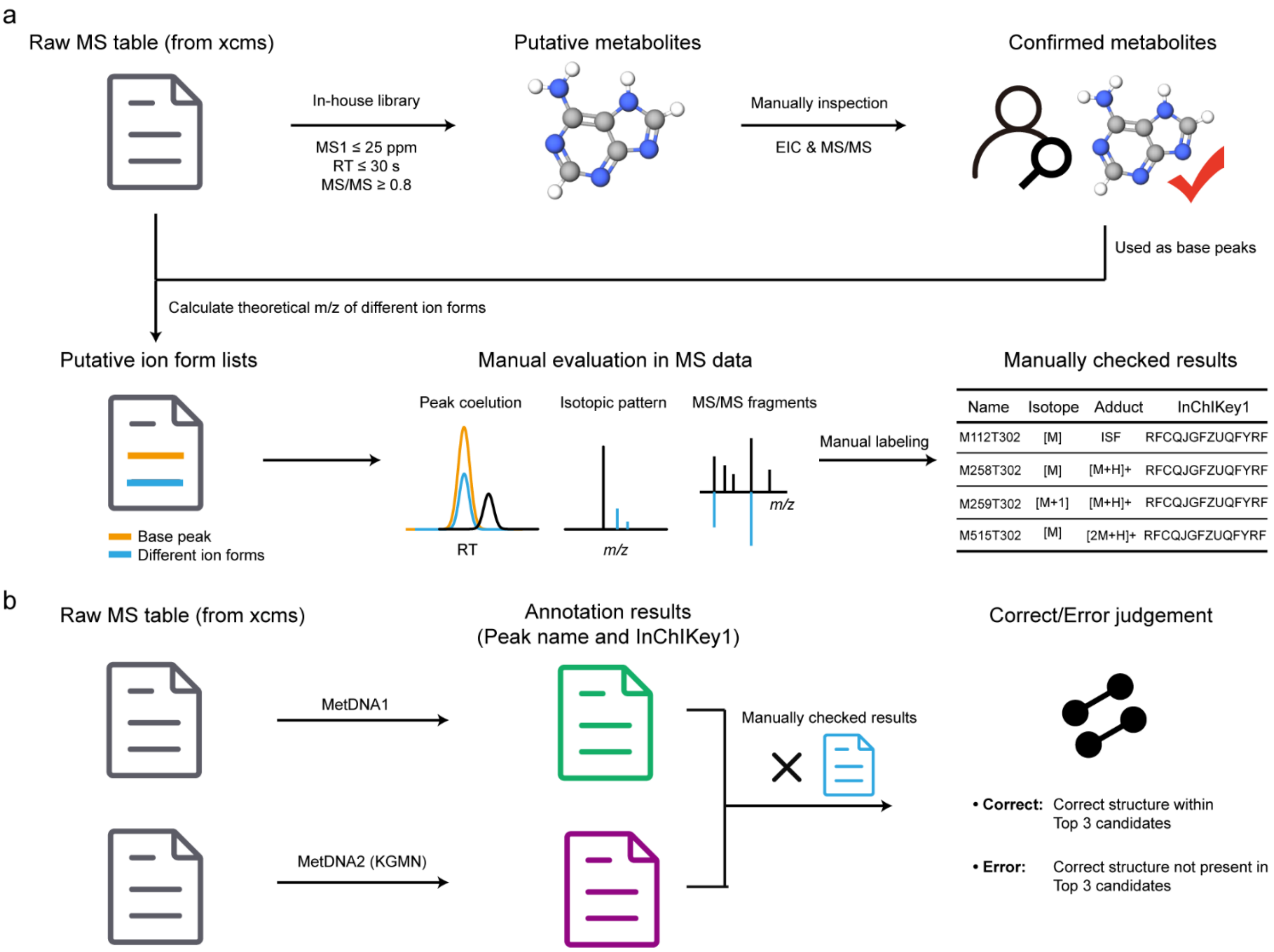

Supplementary Figure 5. The workflow of accuracy evaluation with a manually curated data set. (**a**) Curation of manually checked table; (**b**) Comparison of annotation results with manually checked results.

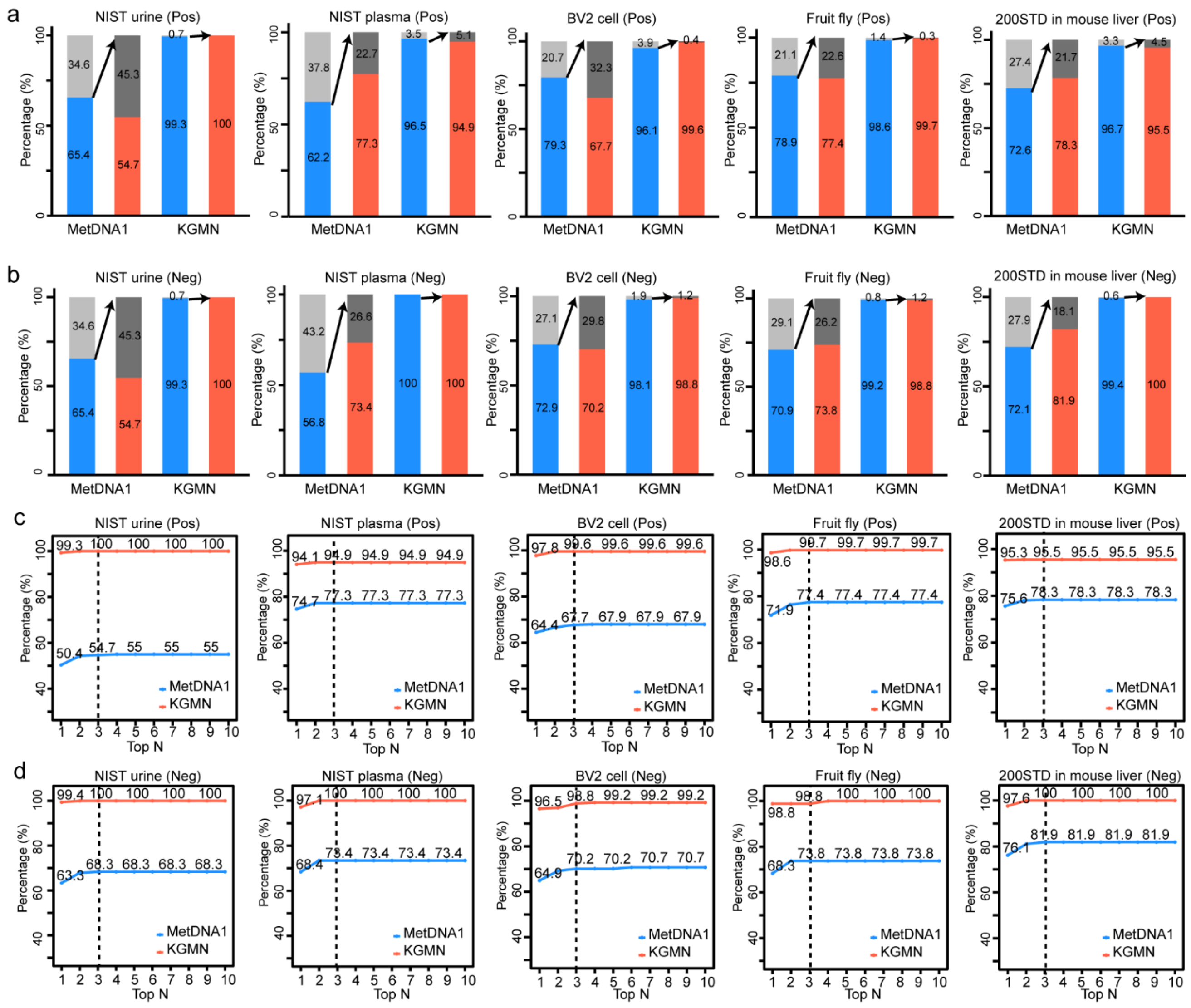

Supplementary Figure 6. Comparison between MetDNA1 and KGMN in different biological samples, including NIST human urine, NIST human plasma, BV2 cells, head tissues of fruit fly, and 200STD spiked mouse liver tissues. (**a-b**) Comparison of annotation coverages and correct/error percentages between MetDNA1 and KGMN in positive (**a**) and negative modes (**b**), respectively. (**c-d**) Correct and error rates among top n (n = 1 to 10) annotations in different biological samples in positive (**c**) and negative modes (**d**), respectively.

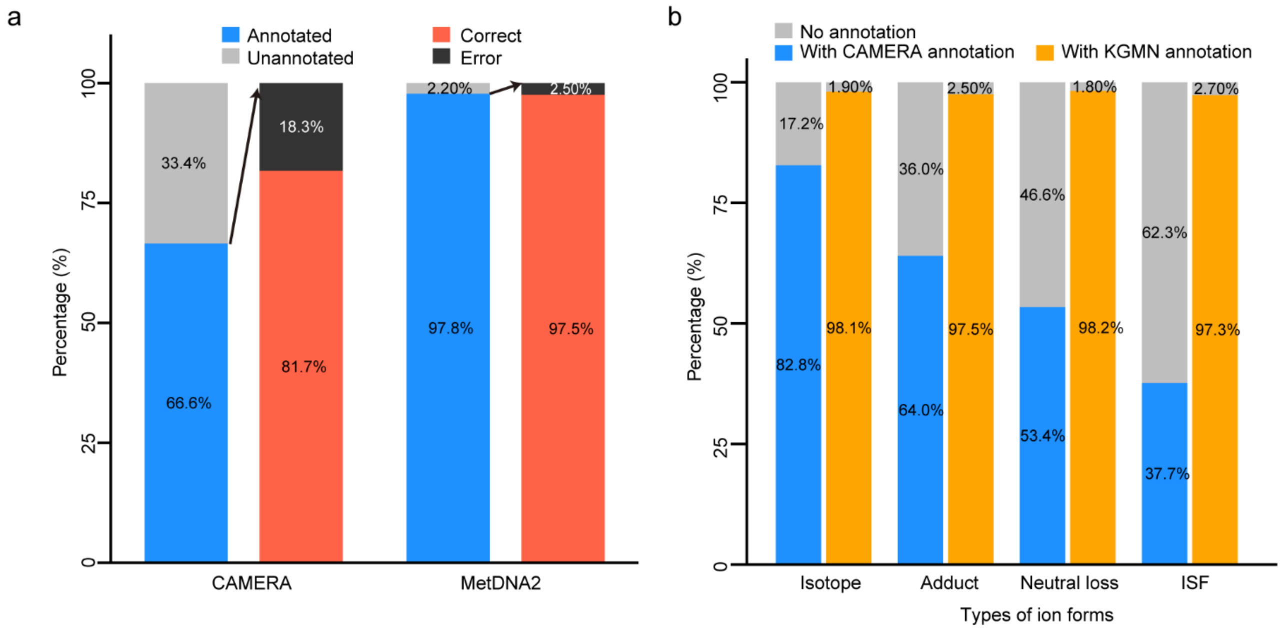

Supplementary Figure 7. Benchmark comparison between CAMERA and KGMN for annotating ion forms of metabolic peaks. (**a**) Percentages of annotation coverage and correct/error rates for annotating ion forms of metabolic peaks. (**b**) Annotation percentages for different types of ion forms. The R package "CAMERA" (v1.46.0) and the same rule table were used for evaluation.

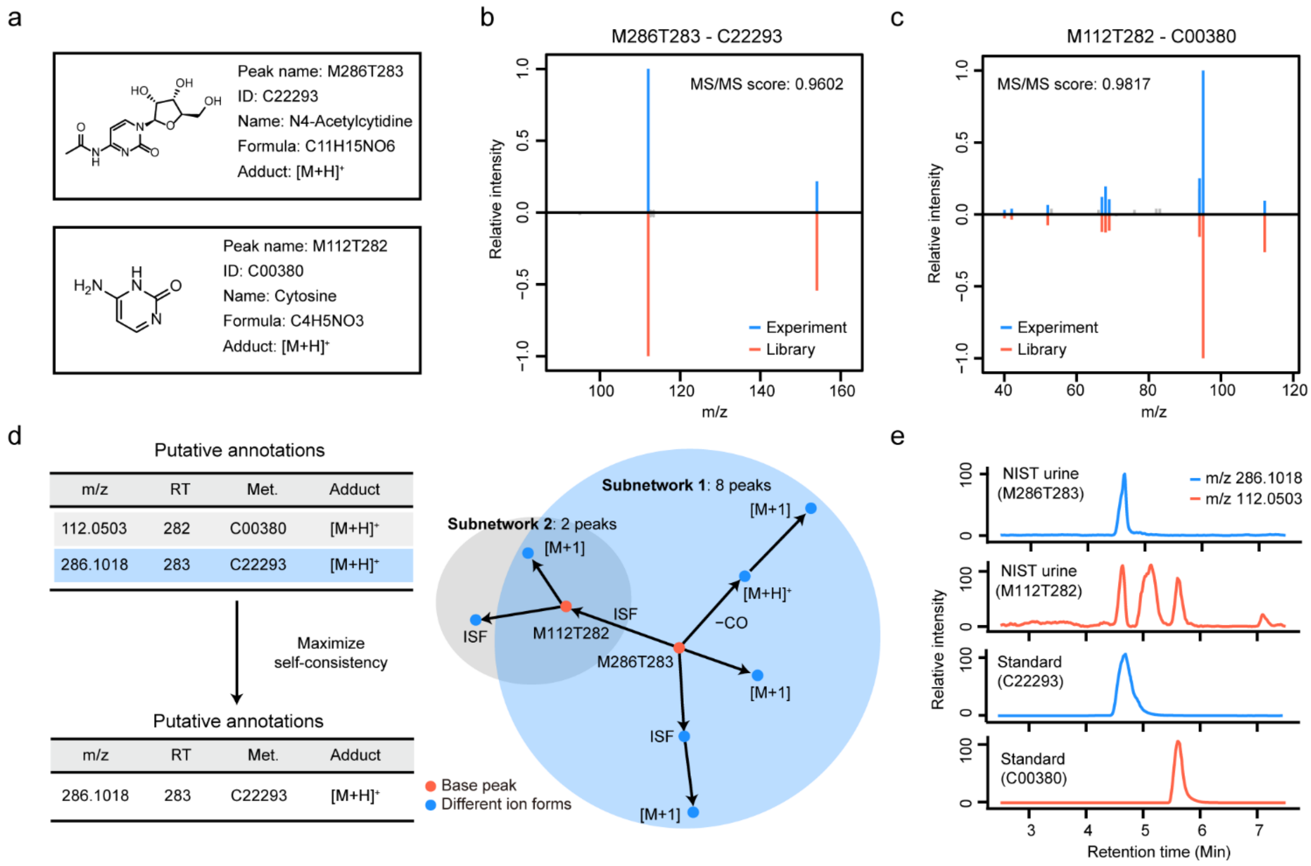

Supplementary Figure 8. KGMN recognized the in-source fragments of N4-Acetylcytidine. (**a**) Peak M286T283 and peak M112T282 were annotated as N4-Acetylcytidine and cytosine in MetDNA1, respectively. (**b-c**) MS/MS spectral match between experimental MS/MS spectra and the standard spectral libraries for N4-Acetylcytidine (**b**) and cytosine (**c**). (**d**) Peak correlation subnetwork recognized M112T282 as an in-source fragment of M286T283. (**e**) The parallel acquisition of NIST human urine sample and chemical standards confirmed that peak M112T282 is an in-source fragment of M286T283.

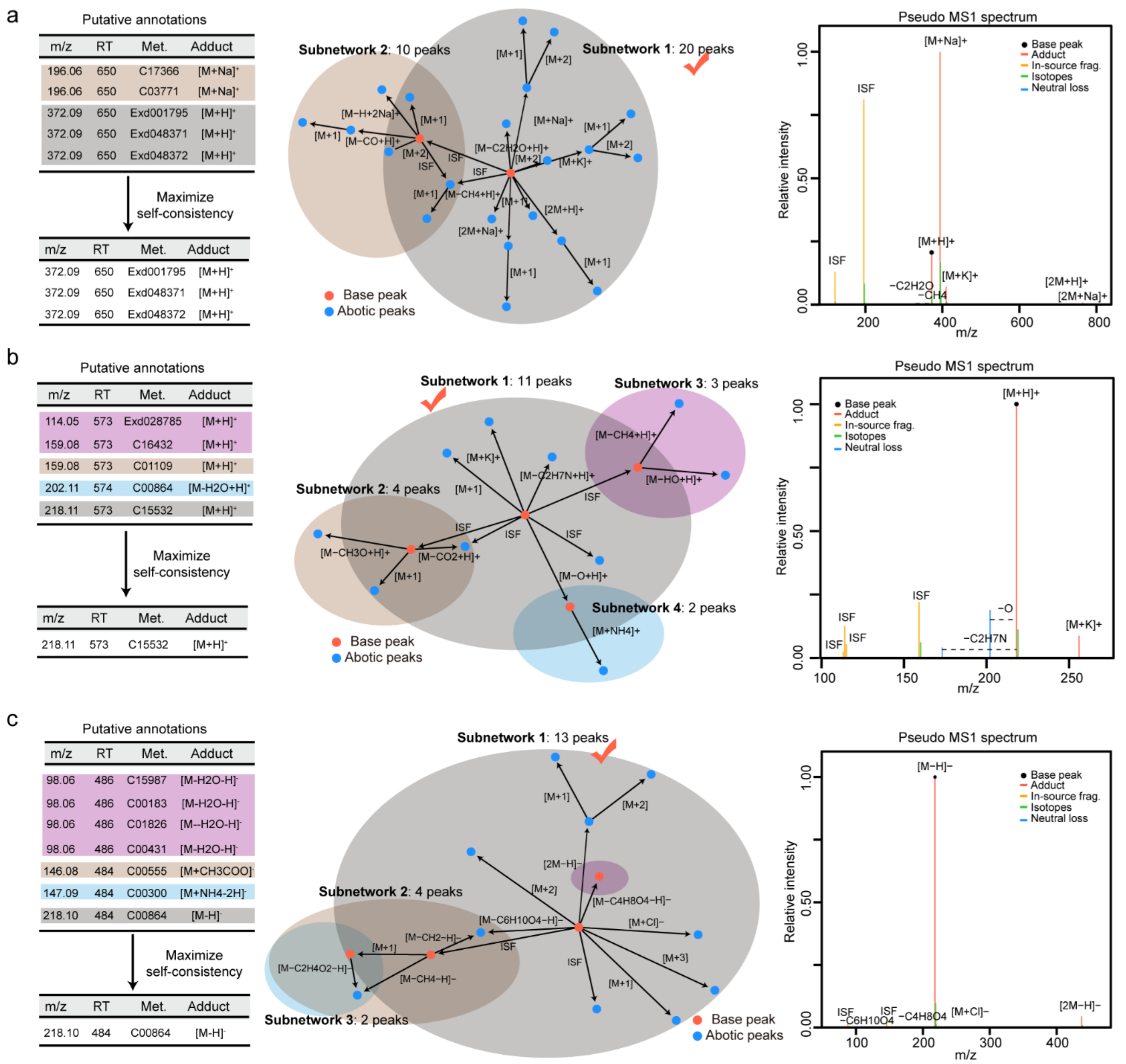

Supplementary Figure 9. Examples of different ion form recognition and peak assignment in KGMN. (**a-c**) Different ion form peaks and putative annotations for (**a**) M372T650, (**b**) M218T573 and (**c**) M218T484. The left panel is the table for the reduction of putative annotations; the middle panel is the conflicted peak correlation subnetworks; the right panel is the pseudo MS1 spectrum after resolving the conflict peak correlations. The examples were retrieved from NIST human urine samples.

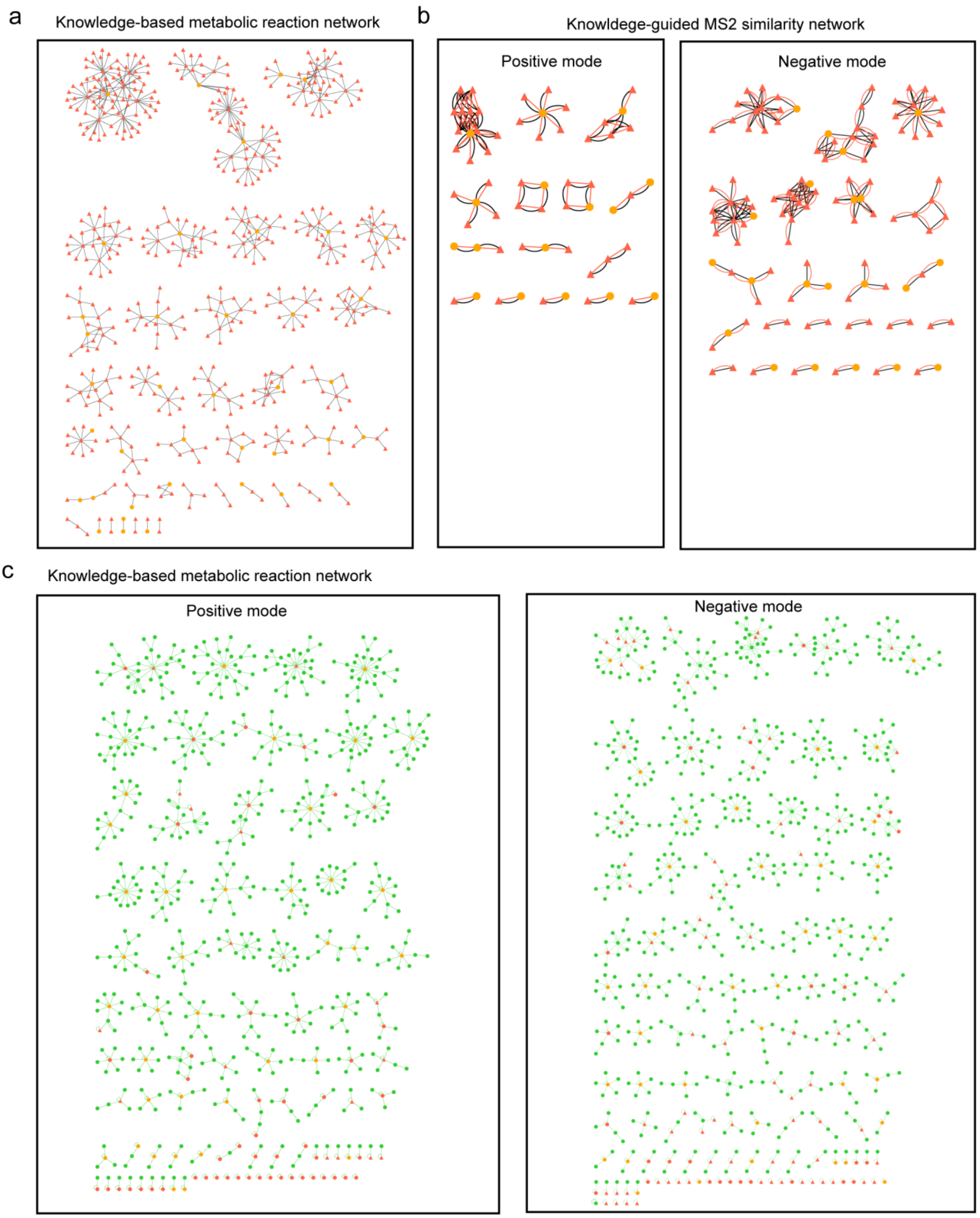

Supplementary Figure 10. Knowledge-guided multi-layer networks of 46std_mix data sets. (**a**) Knowledge-based metabolic reaction network of 46 seed metabolites and unknown metabolites. The orange and red nodes represent seed and unknown metabolites, respectively. The unknown metabolites were curated via *in-silico* enzymatic reactions. The edges represent a biotransformation from known reactions or *in-silico* reactions. This network contains 531 unknown structures and 642

reaction pairs. (**b**) Knowledge-guided MS2 similarity network of 46 seed metabolites and unknown metabolites. The black and red edges represent the biotransformation and MS/MS spectral similarity. The edge of biotransformation represents two nodes can be transformed within 3-step reactions. The edge of MS/MS spectral similarity represents two nodes having MS/MS similarity (dot product score ≥0.5) or shared fragments (n≥4). Only linked peaks are showed here. (**c**) Global peak correlation network of 46std_mix data sets. The orange, red and green nodes represent seed, unknown and different ion form peaks. The edge represents an ion form relationship (isotope, adduct, neutral loss or in-source fragment) between two nodes. A total of 700 and 741 peaks are included in positive and negative modes, respectively.

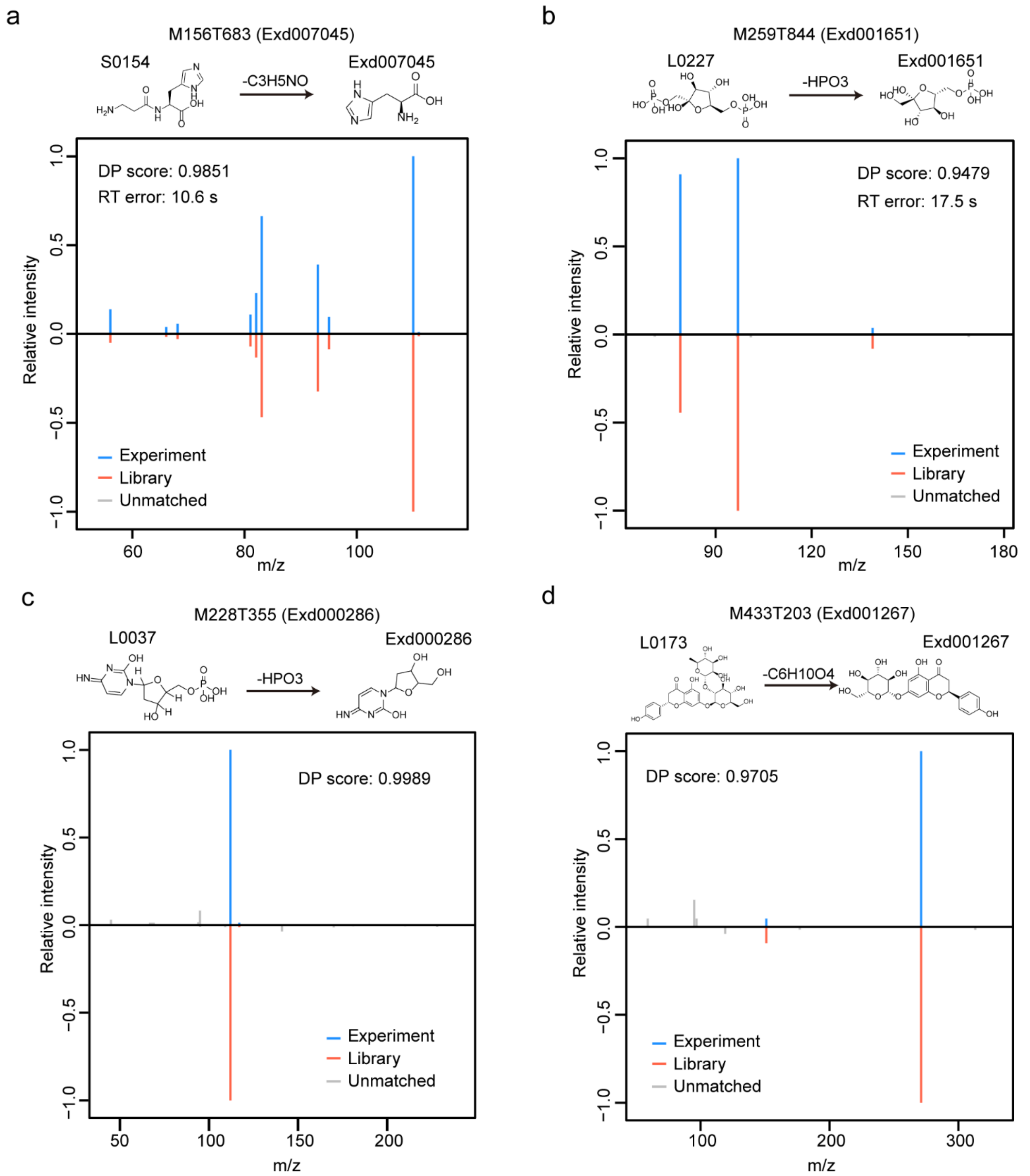

Supplementary Figure 11. Validation examples of annotated unknowns in 46std_mix data sets. (**a-b**) Validation of unknowns using standards: (**a**) M156T683 (Exd007045, L-Histidine); and (**b**) M259T844 (Exd001651, D-Fructose 6-phosphate) in positive and negative modes, respectively; (**c-d**) validation of unknowns using public spectral databases: (**c**) M228T355 (Exd000286, Deoxycytidine), and (**d**) M433T203 (Exd001267, Naringenin 7-O-beta-D-glucoside) through MoNA and Metlin databases, respectively.

a

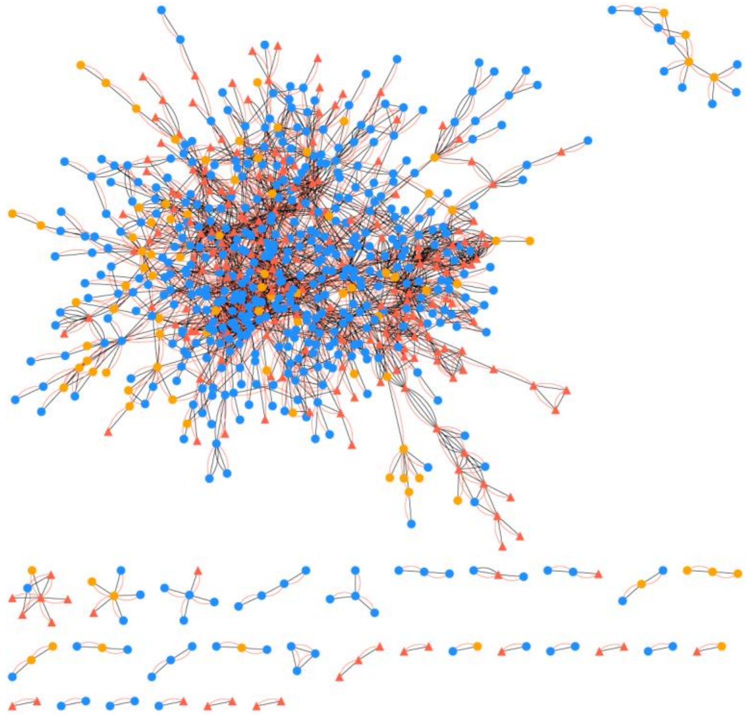

b

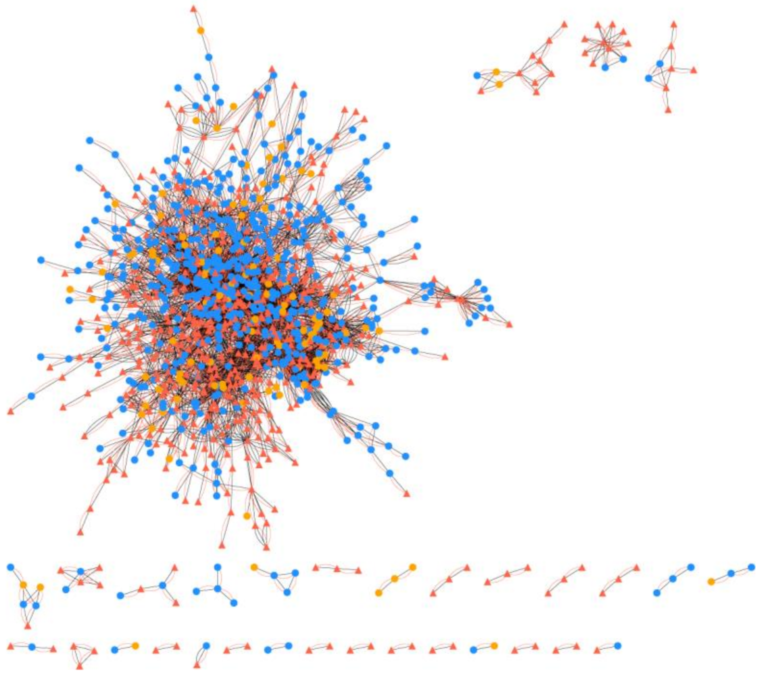

Supplementary Figure 12. Knowledge-guided MS/MS similarity network of NIST human urine sample: (**a**) positive mode; (b) negative mode. The positive mode network contains 1,100 nodes, and 3,170 edges. The negative mode contains 1,444 nodes, and 7,810 edges. The orange, blue, and red nodes represent seed, known and unknown metabolites, respectively. The black and red edges represent the biotransformation edge and the MS/MS similarity edge, respectively. The edge of biotransformation represents two nodes can be transformed within 3-step reactions. The edge of MS/MS similarity represents two nodes having MS/MS similarity (dot product score ≥ 0.5) or shared fragments (n ≥ 4). Only linked peaks are showed here.

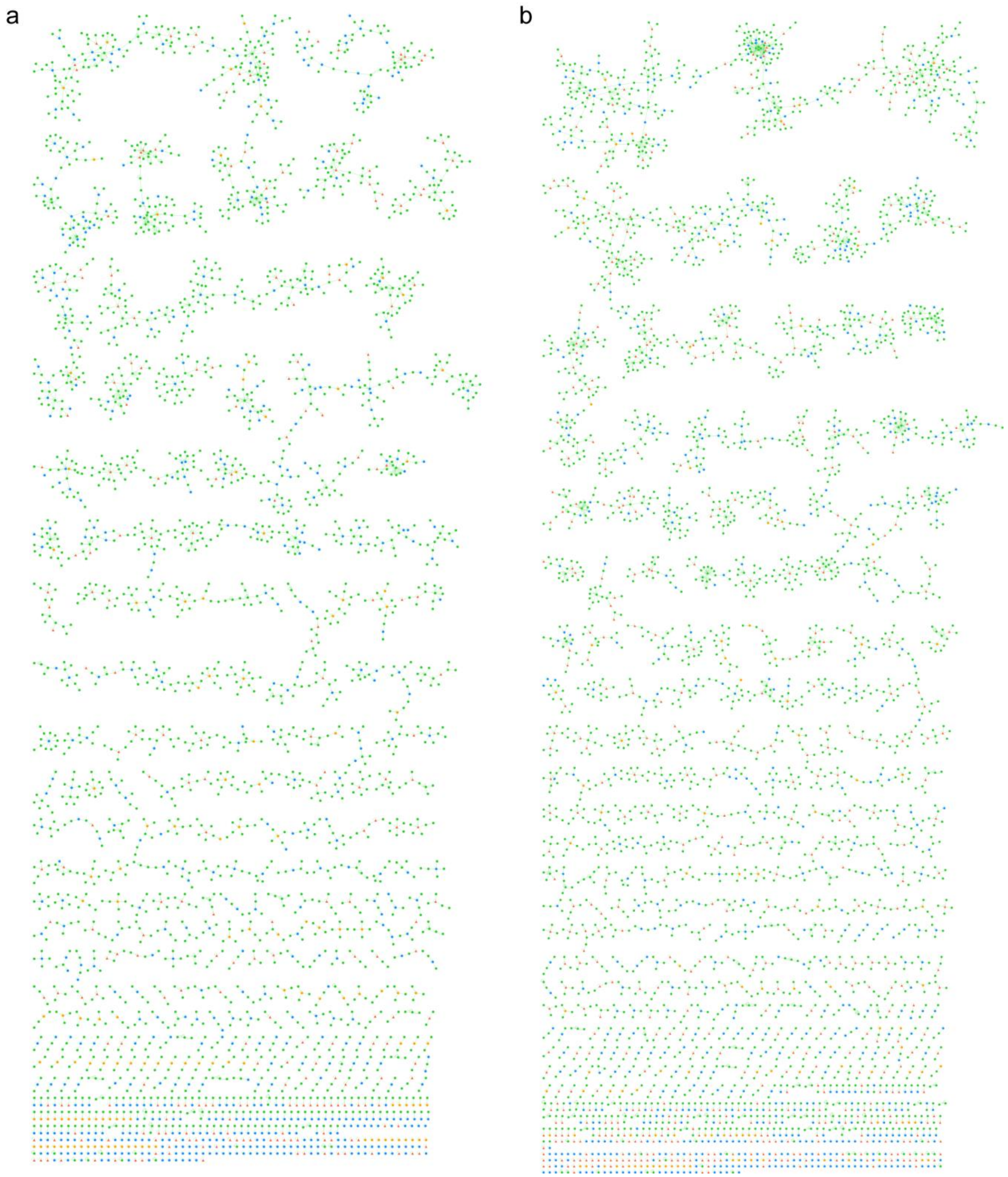

Supplementary Figure 13. Global peak correlation network of NIST human urine sample in positive (**a**) and negative (**b**) modes. It contains 3,301 nodes and 4,374 edges in positive mode, and 4,117 nodes and 5,750 edges in negative mode. The orange, blue, and red nodes represent seed, known and unknown metabolites from network 2, which were used as base peaks here. The green nodes represent different ion form peaks.

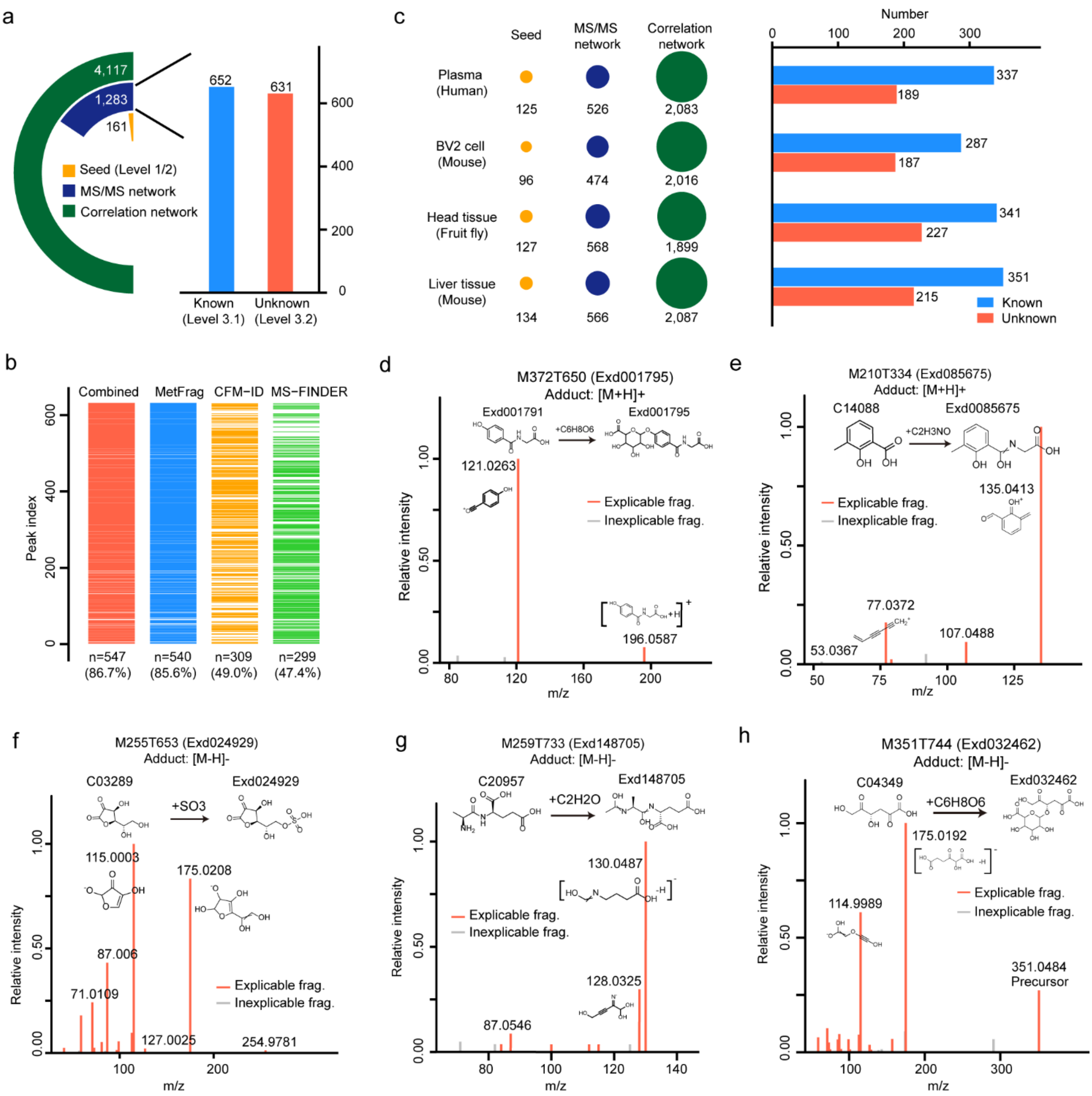

Supplementary Figure 14. Global annotation of unknown metabolites in negative mode and validation examples of unknowns using *in-silico* MS/MS tools. (**a**) The annotated known and unknown metabolites in NIST human urine samples in negative mode. The left panel is the statistics of annotated peaks in the multi-layer networks, and the right panel is the statistics of annotated known and unknown peaks. (**b**) Validations of annotated unknown metabolites in urine samples with different *in-silico* MS/MS tools. (**c**) Global annotations of metabolites in different biological samples in negative mode. The left panel is the statistics of annotated peaks in the multi-layer network, and the right panel is the statistics of known and unknown metabolites. (**d-h**) Validation examples of unknown metabolites using *in-silico* MS/MS tools.

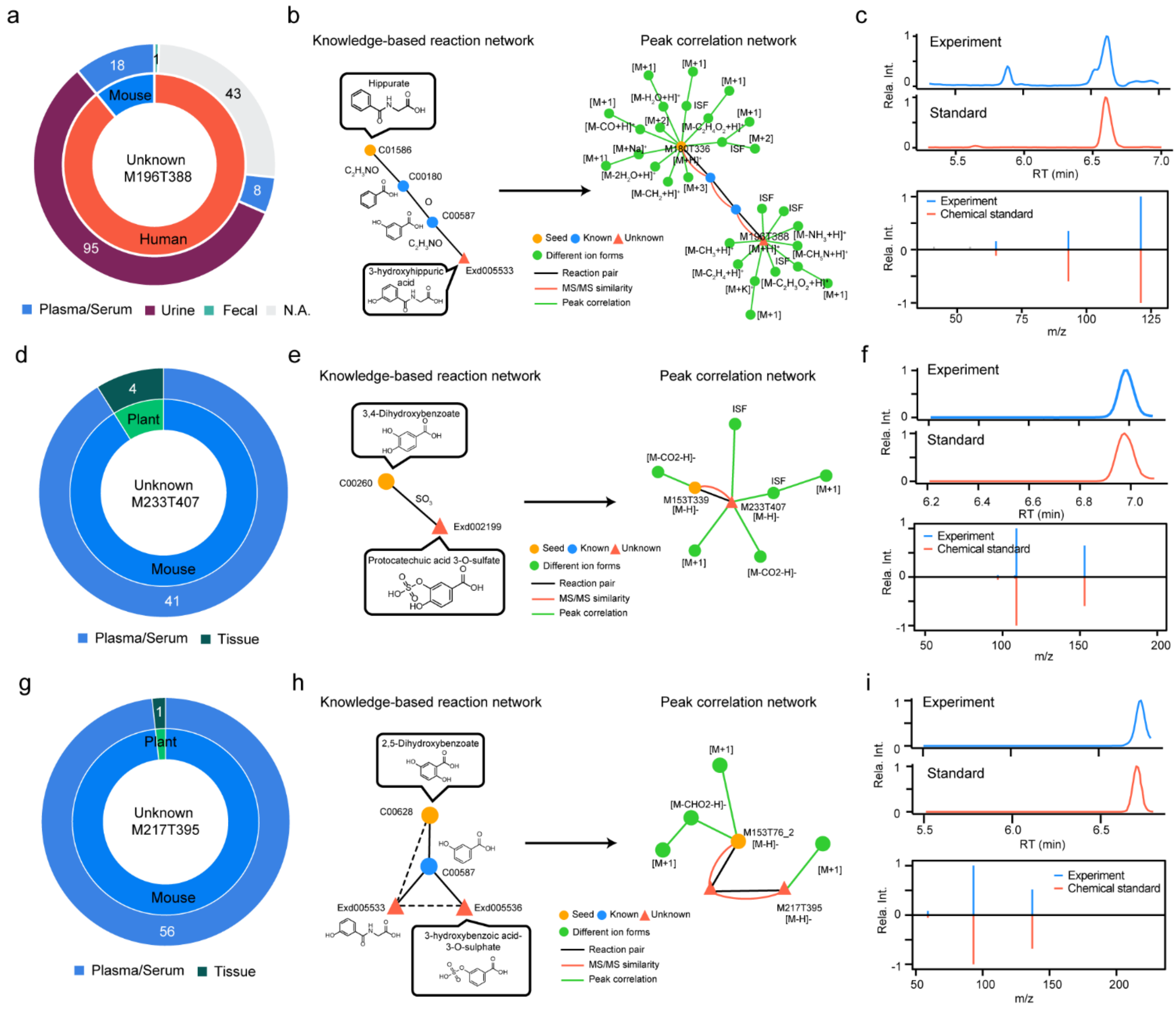

Supplementary Figure 15. The repository-mining and structural validations of 3 recurrent unknown metabolites. (**a-c**) a recurrent unknown metabolite (M196T388, 3-hydroxyhippuric acid); (**d-g**) a recurrent unknown metabolite (M233T407, protocatechuic acid 3-O-sulfate); (**g-i**) a recurrent unknown metabolite (M217T395, 3-hydroxybenzoic acid-3-O-sulphate). The panels **a**, **d**, **g** are recurrent distributions of species and sample types; the inner and outer pie plots are the distributions in species and sample types, respectively. The panels **b**, **e**, **h** are the details of unknown annotations using KGMN. The panels **c**, **f**, **i** are the structural validations using the synthetic standards by matching the retention time and MS/MS spectra.

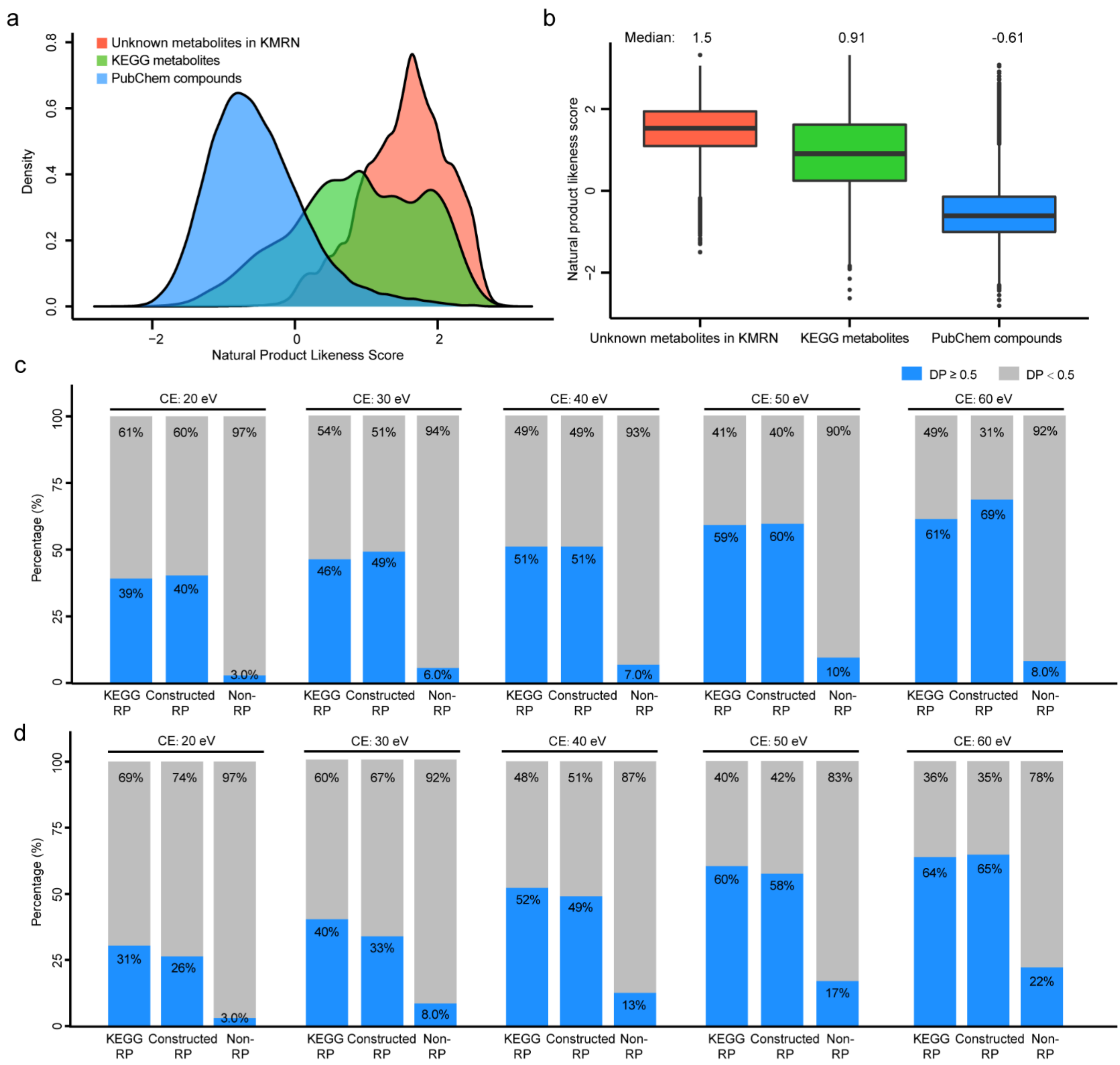

Supplementary Figure 16. Curated unknown metabolites and reaction pairs in the knowledge-based metabolic reaction network (KMRN). (**a**) Distribution of natural product likeness score of unknown metabolites in KMRN, KEGG metabolites, and PubChem compounds. 100,000 PubChem compounds were randomly retrieved to represent the PubChem database. (**b**) Natural product likeness score of unknown metabolites in KMRN (n=159,083), KEGG metabolites (n=16,085), and PubChem compounds (n=100,000). (**c**-**d**) MS/MS spectral similarity comparison among KEGG reaction pairs, *in-silico* curated unknown reaction pairs (i.e., constructed RP), and non-reaction pairs in positive (**c**) and negative (**d**), respectively. The lower, middle and upper lines in box plots (**b**) correspond to 25th, 50th and 75th quartiles, and the whiskers extend to the most extreme data point within 1.5 interquartile range (IQR).

Supplementary Table 1. The supported data processing tools with KGMN

Stage	Usage	Tool	Version	Tutorial
Peak picking software	Generation of required feature list for KGMN	XCMS	V1.46.0 or higher	Link 1
		MS-DIAL	V4.60 or higher	Link 2
		MZmine	V3.0.21 or higher	Link 3
In-silico MS/MS tools	Cross validation of putative metabolites from KGMN	MetFrag	V2.4.5 or higher	Link 4
		CFM-ID	V2.4 or higher	Link 4
		MS-FINDER	V3.24 or higher	Link 4
Repository mining	Search in the metabolomics repository	MASST	Workflow29	Link 5
Visualization	Visualization of KGMN results	Cytoscape	V5.8.3 or higher	Link 6

Note:

- Link 1: http://metdna.zhulab.cn/metdna/help#3.1
- Link 2: http://metdna.zhulab.cn/metdna/help#3.2
- Link 3: http://metdna.zhulab.cn/metdna/help#3.3
- Link 4: https://github.com/ZhuMetLab/MetDNA2_Web/blob/main/Tutorials/Tutorial_KGMN_and_insilico_ms2.pdf
- Link 5: https://github.com/ZhuMetLab/MetDNA2_Web/blob/main/Tutorials/Tutorial_KGMN_and_MASST.pdf
- Link 6: https://github.com/ZhuMetLab/MetDNA2_Web/blob/main/Tutorials/Tutorial_visualization.pdf

Supplementary Table 2. Statistics of global peak annotation optimization to improve annotation accuracy.

No.	Data set (Polarity)	Peaks	MetDNA1			MetDNA2		
			Peak with candi.	Candi.	Accuracy (Top3)	Peak with candi.	Candi.	Accuracy (Top 3)
1	NIST urine (Pos)	425	278	596	152 (54.7%)	422	464	422 (100%)
2	NIST urine (Neg)	325	221	423	151 (68.3%)	313	316	313 (100%)
3	NIST plasma (Pos)	368	229	361	177 (77.3%)	355	392	337 (94.9%)
4	NIST plasma (Neg)	139	79	129	58 (73.4%)	139	153	139 (100%)
5	BV2 cell (Pos)	464	368	604	249 (67.7%)	446	457	444 (99.6%)
6	BV2 cell (Neg)	262	191	307	134 (70.2%)	257	286	254 (98.8%)
7	Fruit fly head (Pos)	365	288	442	223 (77.4%)	360	383	359 (99.7%)
8	Fruit fly head (Neg)	258	183	353	135 (73.8%)	256	280	253 (98.8%)
9	200STD in mouse liver (Pos)	508	369	459	289 (78.3%)	491	506	469 (95.5%)
10	200STD in mouse liver (Neg)	337	243	361	199 (81.9%)	335	356	335 (100%)
Summary		3,451	2,449	4,035	1,767	3,374	3,593	3,325

Supplementary Table 3. Statistics of biotransformation types in 46std_mix data set.

No.	Biotransformation	Positive mode	Negative mode
1	C6H8O6	6	47
2	SO3	10	44
3	HPO3	24	30
4	O	7	17
5	H2O	3	11
6	C2H2O	0	4
7	CH3	0	4
8	C2H3NO	0	3
9	C4H4O3	1	2
10	C3H5NO	2	1
11	C10H10N4O3	0	1
12	C6H10O4	0	1
13	C6H9O6	0	1
14	H2	0	1
15	CO2	1	0

Supplementary Table 4. Statistics of annotated peaks in different biological samples

Data sets	Seed peaks	MS/MS network			Peak correlation network
		Known	Unknown	Sum	
NIST urine (Pos)	173	634	293	927	3,301
NIST urine (Neg)	161	652	631	1,283	4,117
NIST plasma (Pos)	135	310	73	383	1,774
NIST plasma (Neg)	125	337	189	526	2,083
BV2 cell (Pos)	188	398	183	581	2,827
BV2 cell (Neg)	96	287	187	474	2,016
Fruit fly brain (Pos)	187	265	122	387	1,883
Fruit fly brain (Neg)	127	341	227	568	1,899
Mouse liver (Pos)	209	270	107	377	2,464
Mouse liver (Neg)	134	351	215	566	2,087
Average	154	385	223	607	2,445

Supplementary Table 5. Statistics of unknown biotransformation types in NIST urine data set

No.	Biotransformation	Pos	Neg	No.	Biotransformation	Pos	Neg
1	SO3	322	1045	31	H	2	3
2	C6H8O6	353	905	32	C19H20N3O11P	2	2
3	H2	251	505	33	C29H49N3O17P2	0	2
4	O	71	160	34	C3H3O5P	0	2
5	HPO3	15	119	35	C5H8NO3	0	2
6	H2O	100	108	36	CO	6	2
7	C2H2O	60	105	37	C12H22N2O7	0	1
8	C2H3NO	57	83	38	C14H26O	0	1
9	CH2	41	59	39	C15H9O4	4	1
10	isomer	33	56	40	C18H14N2O7	0	1
11	CH3	10	38	41	C2H4O	0	1
12	C7H12O6	20	34	42	C30H25O12	6	1
13	C6H9O6	3	29	43	C30H48O2	1	1
14	C6H10O5	33	22	44	C3H2O	0	1
15	C6H11O5	17	20	45	C61H100O11P2	0	1
16	CO2	10	19	46	C67H110O16P2	0	1
17	C11H18O10	0	10	47	C6H13N4O	0	1
18	C7H10O6	8	10	48	C8H13NO	0	1
19	C2O3	0	6	49	HO3S	0	1
20	C15H9O5	3	4	50	-2O+H	12	0
21	C23H34N4O19P2	7	4	51	C3H2O3	6	0
22	C2H4	2	4	52	C33H50O8	5	0
23	C5H7NO3	0	4	53	C27H40O2	3	0
24	C10H15N3O6S	2	3	54	C6H10O4	2	0
25	C12H16O10	3	3	55	C7H4O4	1	0
26	C12H20O10	5	3	56	C7H5NO	1	0
27	C15H8O2	6	3				
28	C3H6NO	1	3				
29	C8H12O7	4	3				
30	CH6N7O15P3S	0	3				

Supplementary Note 1.

Tutorial of KGMN result visualization and analysis

Zhiwei Zhou

2022-06-05

Introduction

Unknown metabolite annotation is one of long-standing challenges in untargeted metabolomics. We develop an approach, namely, knowledge-guided multi-layer network (KGMN), to enable global metabolite annotation from knowns to unknowns in untargeted metabolomics. The KGMN approach integrates three-layer networks, including knowledge-based metabolic reaction network (Network 1), knowledge-guided MS/MS similarity network (Network 2), and global peak correlation network (Network 3). This tutorial will help users to visualize, reproduce and investigate putatively annotated known and unknown metabolites from KGMN.

1. Installation

The analysis and visualization of KGMN results mainly relies on R package – MetDNA2Vis, and its depended R packages; The Cytoscape software is used for manually visualize networks, and interactively investigate results of KGMN; The ChemDraw software is involved for drawing chemical structures.

- Install R packages

```
# Install related packages
if(!require(devtools)){
install.packages("devtools")
}

if(!require(BiocManager)){
install.packages("BiocManager")
}

# Install CRAN/Bioconductor packages
required_pkgs <- c("dplyr","tidyr","readr","CHNOSZ","igraph",
  "magrittr","ggplot2","ggraph","tidygraph")
```

```r
list_installed <- installed.packages()

new_pkgs <- required_pkgs[!(required_pkgs %in% list_installed[,'Package'])]
if (length(new_pkgs) > 0) {
  BiocManager::install(new_pkgs)
} else {
  cat('Required CRAN/Bioconductor packages installed\n')
}


# Install ZhuLab packages
devtools::install_github("ZhuMetLab/SpectraTools")
devtools::install_github("ZhuMetLab/MetDNA2Vis")
```

- Cytoscape software (Version 3.8 or higher required): https://cytoscape.org/
- ChemDraw software (Version 19.0 or higher required): https://perkinelmerinformatics.com/products/research/chemdraw

2. Step-by-step instruction for visualization

In this part, we introduce how to visualize multi-layer networks from KGMN. It will help users to reproduce figures in KGMN manuscripts. Here, the Human NIST urine (Positive data, used in KGMN manuscript) is used as a demo dataset. This data set have been processed and exported by **MetDNA2 web server** (version 1.0.4). The raw data files and results can be downloaded at **here** (https://mega.nz/file/8v50iL6T#oILf8wlVJU_iqTfjcOtH1TRHhnP1GGbvG_ZNb1xniGc). The more details of sample extraction and data preprocessing can be found in our KGMN manuscript.

The step-by-step demonstration is provided as below.

2.1 Download demo data and unzip the archive.

- All required intermediate files for visualization is provided in '06_visualization' folder.

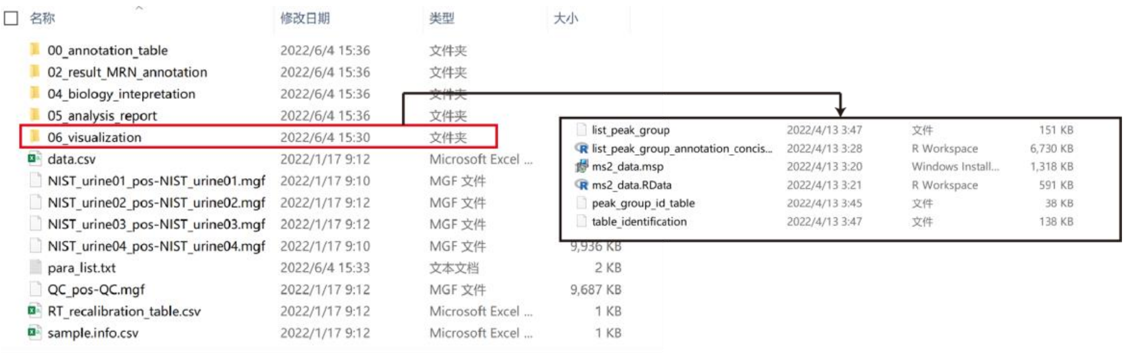

2.2 Preparing.

- Set the working directory ('your_path/06_visualization') and load required packages. Then, please check required files whether existed.

```
# load packages
library(MetDNA2Vis)
library(CHNOSZ)
library(dplyr)

# check required files
checkFiles4Vis()

## Check required files ...
## Check required files: done!
```

2.3 Reconstruct and export global multi-layer networks.

2.3.1 Network 1

The network 1 is the knowledge-guided metabolic reaction network. For knowns, the KEGG reaction pair network is directly used. For unknowns, an extended KEGG reaction pair network is used. The network expansion is performed with in-silico enzymic reactions (via Biotransformer), and further connected with KEGG reaction pair network. The details of network construction and expansion are described in our KGMN manuscript. It should be note that the KEGG reaction pair network and extended network are built in advance.

To export the network 1, it is easily to run reconstructNetwork1 function as below:

```
# export network 1 for visualization
reconstructNetwork1(is_unknown_annotation = TRUE)
```

The networks files will be exported in ‘00_network1’ folder. It contains two files, including “edge_table.tsv” and “node_table.tsv” (Figure 2.3.1). These tables can be import into Cytoscape software for visualization.

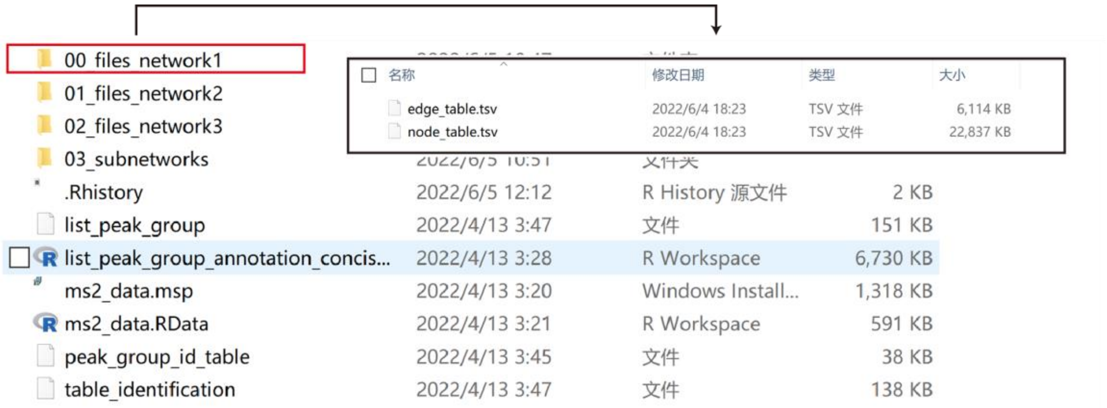

2.3.2 Network 2

The network 2 is a knowledge-guided MS/MS network. Although it calls MS/MS network, differing to MS/MS network (mainly based on MS2), the linkage (edge) of network2 has a prerequisite. It requires a reasonable reaction relationship and definitive structure candidate first. As a result, their retention time can also be predicted. In other words, two linked nodes indicate 4 messages. Their candidates of these nodes have (1) reasonable reaction relationships, (2) low m/z errors, (3) low RT error against with predicted RT values, and (4) MS/MS similarity. It should be note that optimized network2 required to be reconstructed from KGMN exported results, because the global peak correlation network remove and collapse some error nodes and edges in prior analysis. This process usually requires 10-20 min to complete.

To export the network 2, it is easily to run reconstructNetwork2 function as below:

```
# Modify format of KGMN result
annotation_table <- reformatTable1()

# Export global network2 files
reconstructNetwork2(annotation_table = annotation_table,
  is_unknown_annotation = TRUE)
```

The networks files will be exported in '01_network2' folder. The "edge_table.tsv" and "node_table.tsv" in this folder can be imported to Cytoscape.

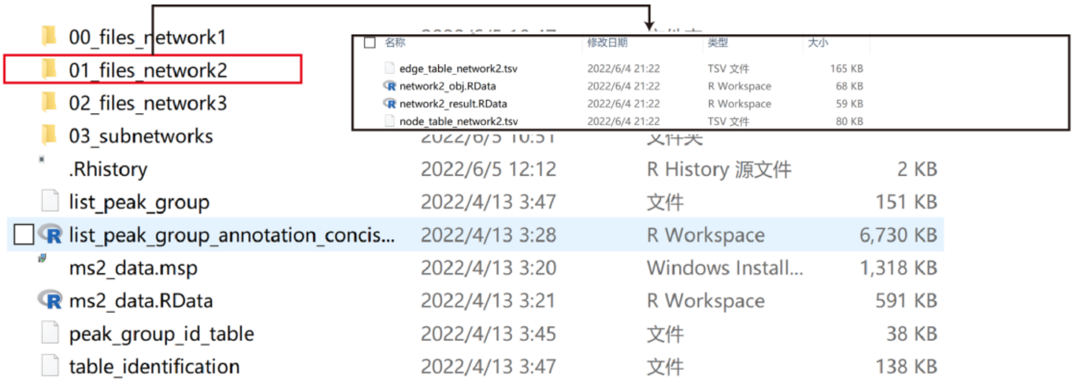

2.3.3 Network 3

The network 3 is the global peak correlation network. This network recognized different ion form peaks derived from peaks from network 2, including adducts, isotopes, neutral losses, and in-source fragments (ISF). The network 3 is used to optimize the annotation and linkage of network 2. The optimization has been completed in KGMN analysis. The details of network 3 construction and optimization can be found in our manuscript.

To export the network 3, it is easily to run reconstructNetwork3 function as below:

```
# export network3
reconstructNetwork3()
```

The networks files will be exported in '**02_files_network3**' folder. The "edge_table.tsv" and "node_table.tsv" in this folder can be imported to Cytoscape for visualization.

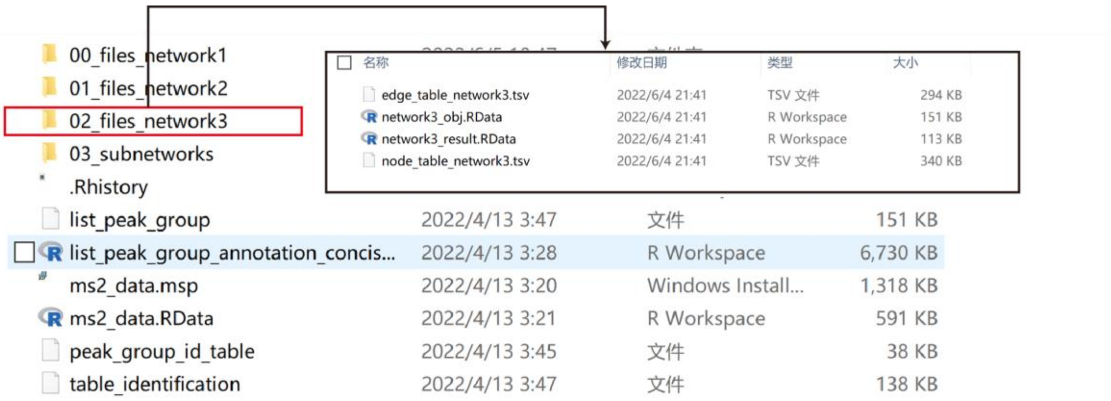

2.4 Visualize global networks with Cytoscape

Above networks (Network 1-3) can be imported to Cytoscape software tool for visualization. The process of network visualization is generally similar. Here, we use the above network 1 as a demonstration. The version of Cytoscape used here is 3.8.2.

Below is the step-by-step instruction:

1. **Import edge file.** Select the “edge_table.tsv” file and open it in the box.

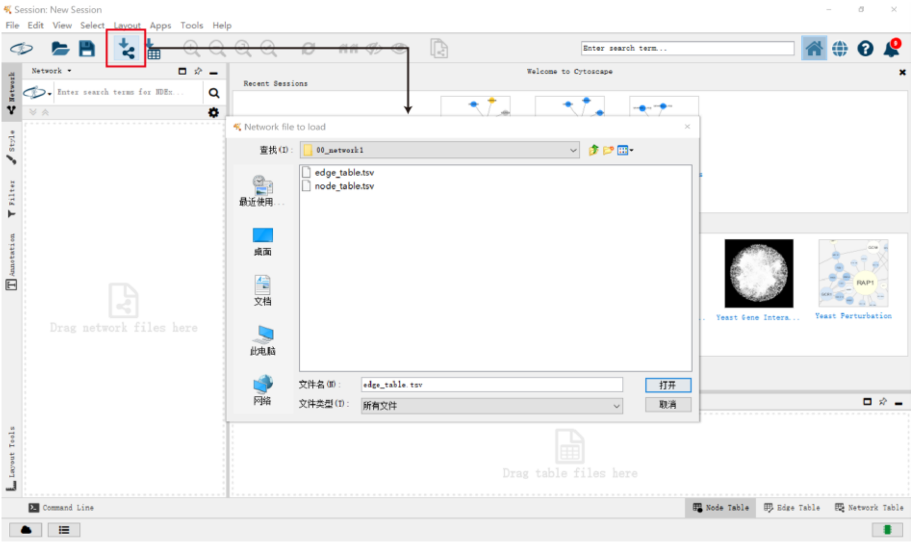

2. **Assign column attributes.** Click the ‘from’ column and select it as “source node”. Similarly, click the “to” column and select it as “target node”. After assigning attributes, click **OK** to construct a network.

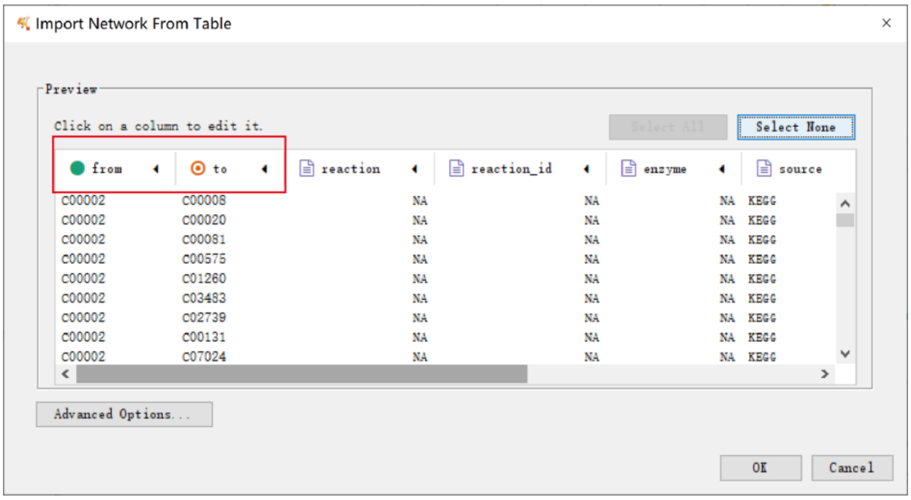

3. **Import node file.** Select the “node_table.tsv” file and open it in the box.

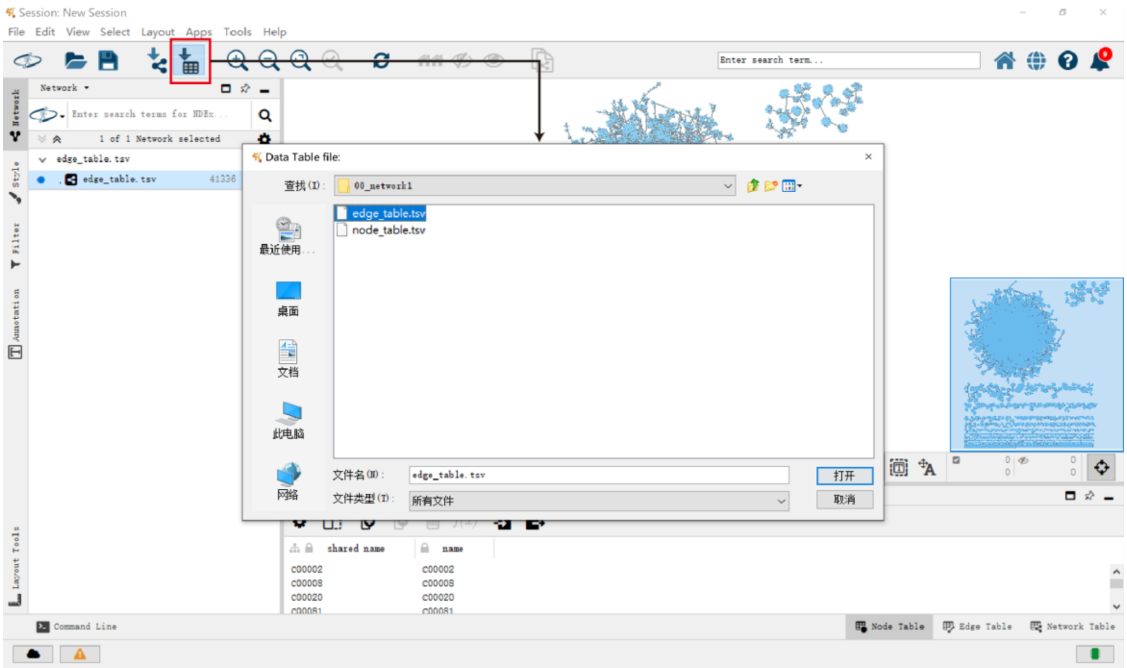

4. Select the “name” column as a key. Then, click the **OK** button.

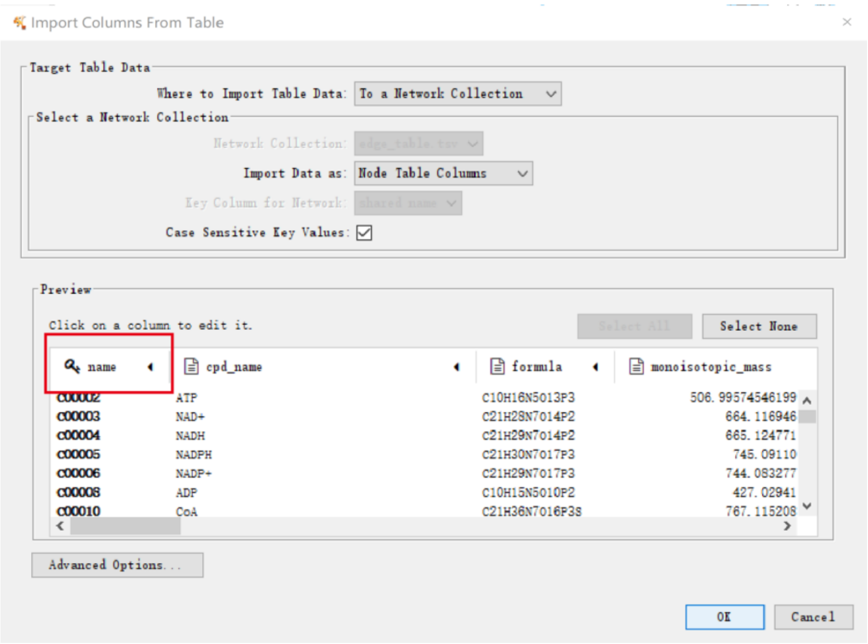

5. **Modify the style for visualization.** Click the Style type, you can adjust node shapes and colors, edge types and colors.

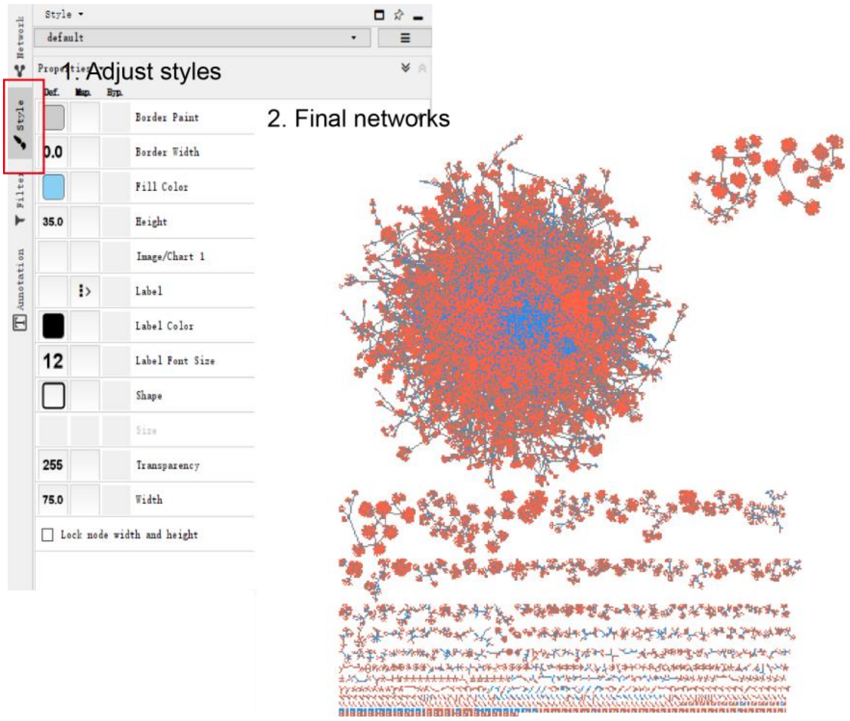

To help users reproduce our plot quickly, users can directly import our style file. The styles of different networks are provided here (https://mega.nz/file/tnp1nKjT#LS1oPzcFzw6bbdsLSqGoW4Qggrl_lM2LsPgsyZXilzQ).

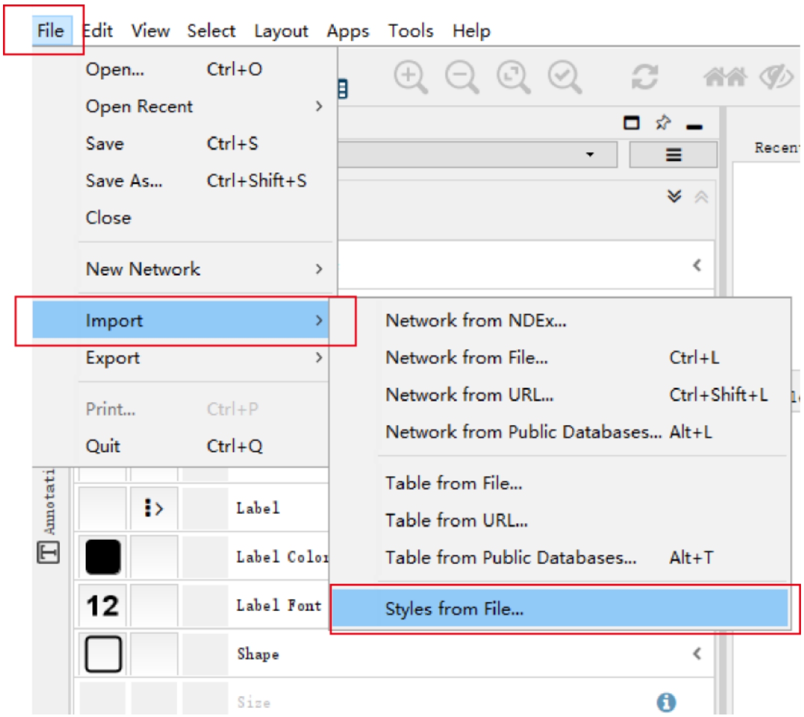

2.5 Select and export interesting subnetwork

Through above procedures, users can easily visualize global network 1-3. With such global networks, users can find interesting subnetworks in Cytoscape. The Cytoscape supports interactively investigation. **It should be note that the targeted subnetwork selection is customized.** Users can directly find interesting nodes from KGMN annotation results, or considering more information, like in-silico MS/MS, chemical structure and/or statistics analysis. For example, in KGMN manuscript, we combined MASST to select an unknown subnetwork of M262T526 (**Figure 5e in manuscript**). This unknown peak was putatively annotated as O-sulfotyrosine, and this annotation was from M182T541-Tyrosine. This subnetwork consisted of 2 peaks and 2 metabolites. Here, we mainly introduce how to export and visualize this subnetwork. First, export network 1 of this subnetwork. **Note:** the export and visualization require intermediate results from global networks. Therefore, please run global peaks export first. To export the subnetwork 1, please directly run `retrieveSubNetwork1` function as below.

```
# network 1 of unknown peak subnetwork
# Note: the folder_output should keep same among different layer subnetworks
retrieveSubNetwork1(centric_met = c('C00082', 'KeggExd000923'),
  is_unknown_annotation = TRUE,
  folder_output = c('M182T541_M262T526'))
```

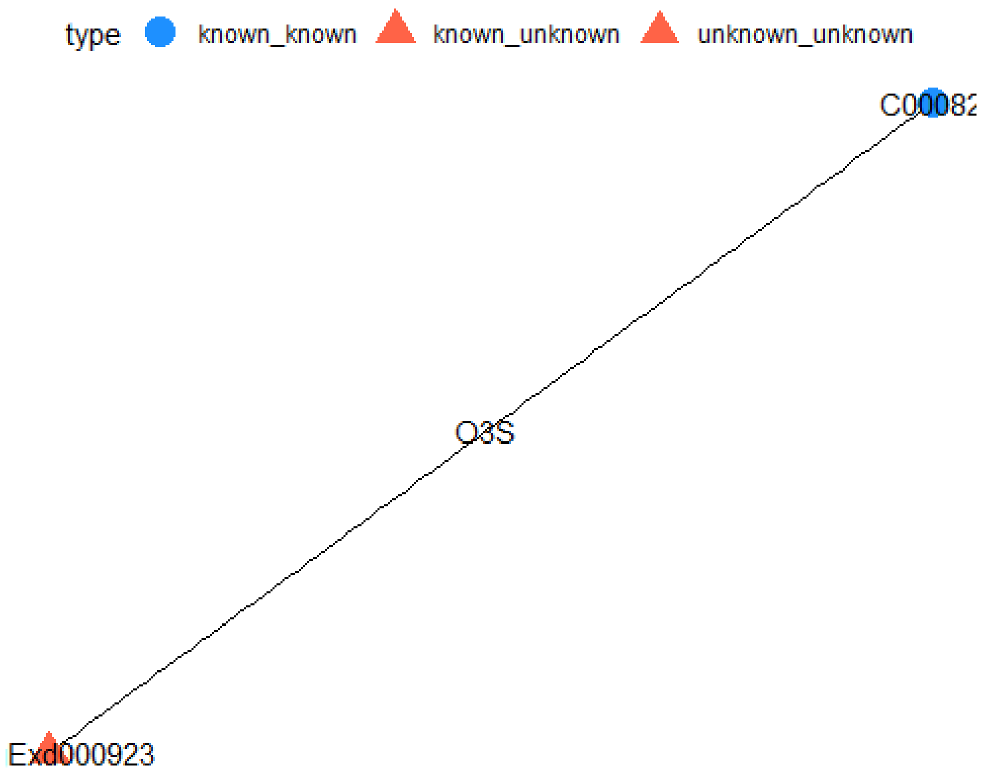

The networks files will be exported in ‘03_subnetworks/your_defined_folder/network 1’ folder. Here, the exported folder is “M182T541_M262T526”. The “edge_table.tsv” and “node_table.tsv” in this folder can be imported to Cytoscape for visualization. **Note:** if you run in RStudio, the preview plot of subnetwork 1 will be directly shown in the plot panel.

Similarly, export network 2 and network 3 of this subnetwork can be completed through running `retrieveSubNetwork2` and `retrieveSubNetwork3` functions, respectively. The preview plots of subnetwork 2 and subnetwork 3 will be shown in the plot panel if you run in RStudio.

```
# network 2 of unknown peak subnetwork
retrieveSubNetwork2(from_peak = 'M182T541',
    end_peak = 'M262T526',
    folder_output = c('M182T541_M262T526'))

## Using `sugiyama` as default layout
```

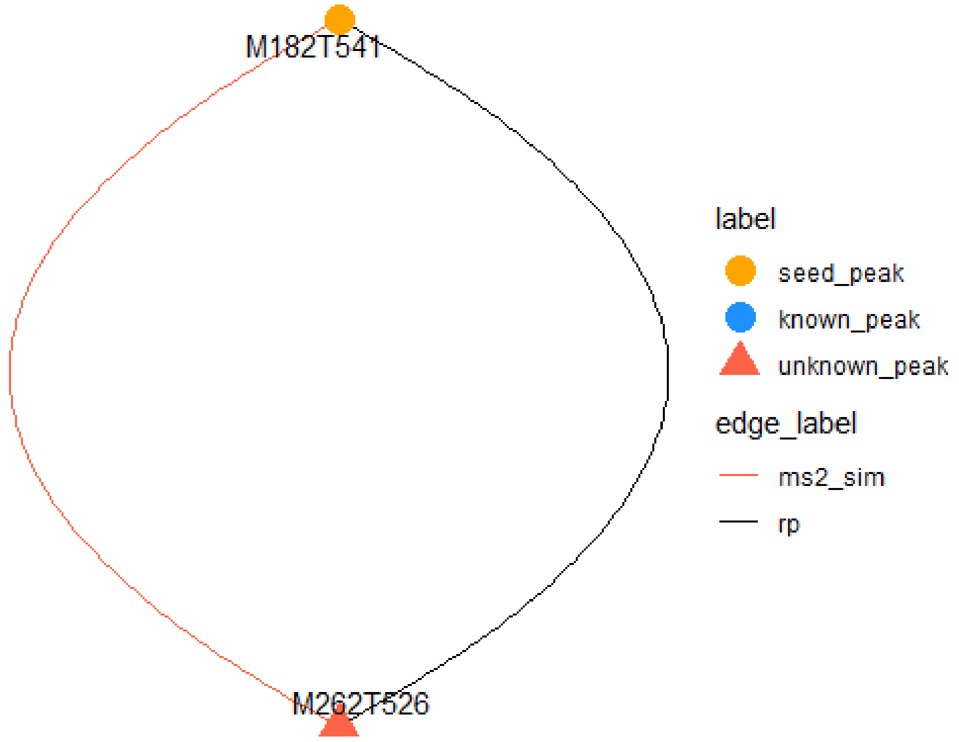


```r
# network 3 of unknown peak subnetwork
retrieveSubNetwork3(base_peaks = c('M182T541', 'M262T526'),
   base_adducts = c('[M+H]+', '[M+H]+'),
   folder_output = c('M182T541_M262T526'))

## Using `stress` as default layout
```

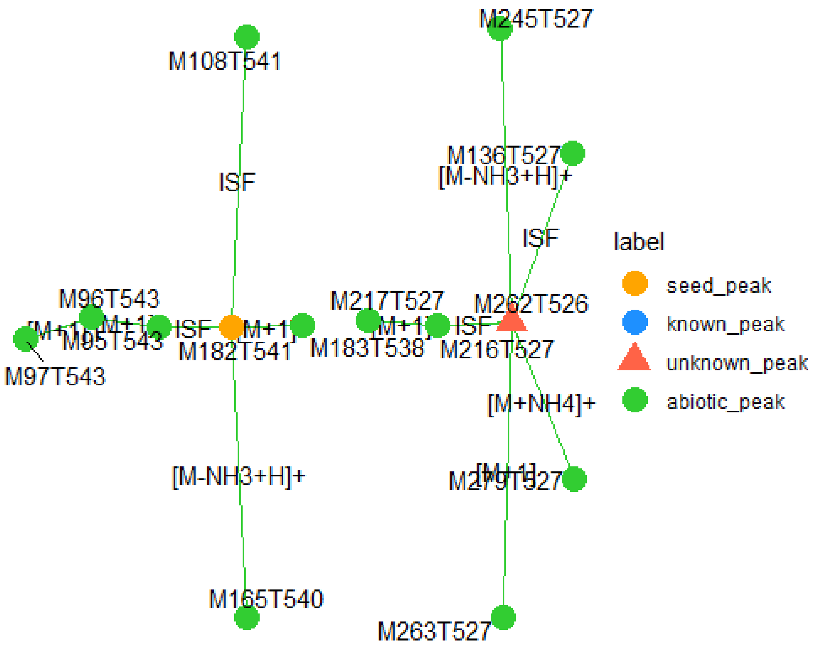

The network 2 and network 3 of the subnetwork can be further merged through running mergeSubnetwork function. The 'network_merge' folder contains node table and edge table for reproduce the merged network.

```
# merge subnetwork
mergeSubnetwork(from_peak = 'M182T541',
  end_peak = 'M262T526',
  folder_output = 'M182T541_M262T526')

## Using `stress` as default layout
```

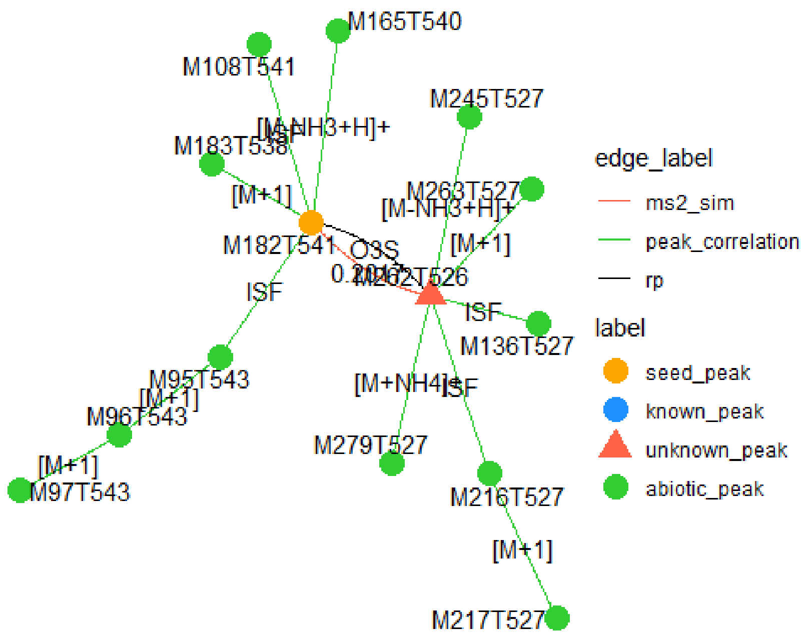

Finally, the folder of subnetwork is organized like below. Each folder contains related files of each network for further visualization in other tools (e.g. Cytoscape).

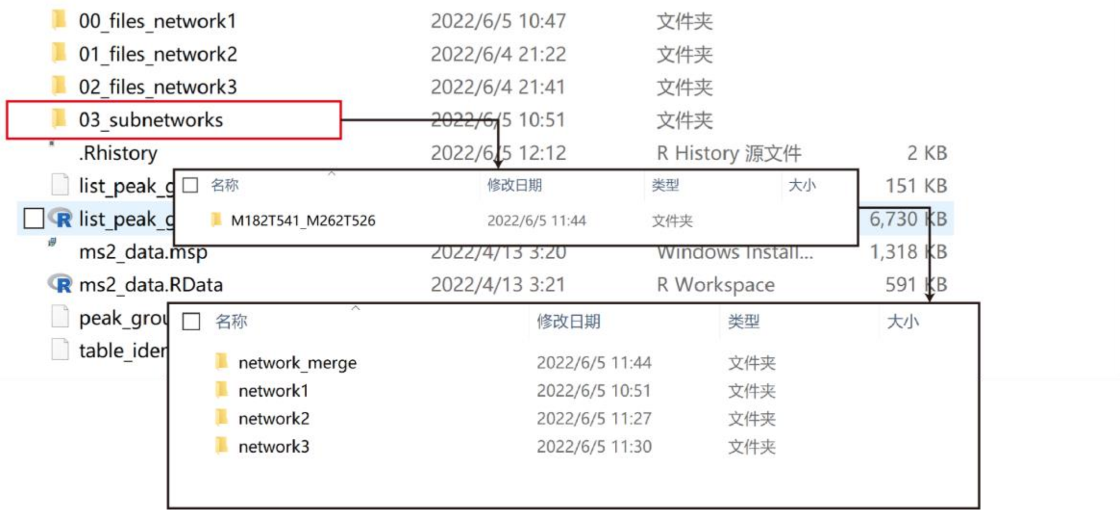

3. The script for visualization

Here is a script which contains above codes to help to reproduce above analysis quickly.

```
# load packages
library(CHNOSZ)
library(dplyr)
library(MetDNA2Vis)

# set working directory
setwd('D:/project/00_zhulab/01_metdna2/00_data/20220602_visualization_kgmn/Demo_MetDNA2_NIST_urine_pos/06_visualization/')

# Export global networks
# construct network 1
reconstructNetwork1(is_unknown_annotation = TRUE)

# construct network 2
annotation_table <- reformatTable1()
reconstructNetwork2(annotation_table = annotation_table)

# construct network 3
reconstructNetwork3()
```

```r
# Export subnetworks ----------------------------------------------------------
# network 1 of unknown peak subnetwork
# Note: the folder_output should keep same among different layer subnetworks
retrieveSubNetwork1(centric_met = c('C00082', 'KeggExd000923'),
    is_unknown_annotation = TRUE,
    folder_output = c('M182T541_M262T526'))


# network 2 of unknown peak subnetwork
retrieveSubNetwork2(from_peak = 'M182T541',
    end_peak = 'M262T526',
    folder_output = c('M182T541_M262T526'))

# network 3 of unknown peak subnetwork
retrieveSubNetwork3(base_peaks = c('M182T541', 'M262T526'),
    base_adducts = c('[M+H]+', '[M+H]+'),
    folder_output = c('M182T541_M262T526'))


# merge subnetwork
mergeSubnetwork(from_peak = 'M182T541',
    end_peak = 'M262T526',
    folder_output = 'M182T541_M262T526')
```

Supplementary Note 2.

Tutorial of validating KGMN unknowns with repository mining

Zhiwei Zhou

2022-06-13

This tutorial aims to help users to select and validate their interesting unknown peaks from KGMN through repository mining. In the manuscript, we mainly used **MASST** to perform repository mining. The MASST[1] is a tool to query spectrum in context of where it occurs against all GNPS data sets. In this tutorial, we focus on demonstrating how to combine KGMN results and MASST. The detail instructions of MASST can be found in **GNPS document** (https://ccms-ucsd.github.io/GNPSDocumentation/masst/).

The step-by-step instruction has been provided below.

1. Data preparing.

In this workflow, the data files require KGMN (MetDNA2) processed firstly. Here, we utilized NIST human urine data set as example. The data set has been analyzed with KGMN (v1.0.4), and the results can be downloaded **here** (https://mega.nz/file/8v50iL6T#oILf8wlVJU_iqTfjcOtH1TRHhnP1GGbvG_ZNb1xniGc).

The folders should look like as below:

Name	Date modified	Type	Size
00_annotation_table	6/6/2022 2:54 PM	File folder	
02_result_MRN_annotation	6/6/2022 2:54 PM	File folder	
04_biology_intepretation	6/4/2022 3:36 PM	File folder	
05_analysis_report	6/6/2022 2:54 PM	File folder	
06_visualization	6/6/2022 2:54 PM	File folder	
data.csv	1/17/2022 9:12 AM	Microsoft Excel C...	2,385 KB
NIST_urine01_pos-NIST_urine01.mgf	1/17/2022 9:10 AM	MGF File	9,877 KB
NIST_urine02_pos-NIST_urine02.mgf	1/17/2022 9:12 AM	MGF File	9,895 KB
NIST_urine03_pos-NIST_urine03.mgf	1/17/2022 9:12 AM	MGF File	9,921 KB
NIST_urine04_pos-NIST_urine04.mgf	1/17/2022 9:10 AM	MGF File	9,936 KB
para_list.txt	6/4/2022 3:33 PM	Text Document	2 KB
QC_pos-QC.mgf	1/17/2022 9:12 AM	MGF File	9,687 KB
RT_recalibration_table.csv	1/17/2022 9:12 AM	Microsoft Excel C...	1 KB
sample.info.csv	1/17/2022 9:12 AM	Microsoft Excel C...	1 KB

The users can browser and select interesting known/unknown peaks in the **annotation table "table1_identification.csv"** in the "00_annotation_table" folder. It should be note that the selection of targeted peak is customized.

For demonstration, we utilized the unknown peak M262T526 as an example (Figure 5d in manuscript). The MS/MS spectrum of this peak can be found in the **"ms2_data.msp"** in "06_visualization" folder. You can open it with text tool (e.g. Notepad++).

```
NAME: M262T526
PRECURSORMZ: 262.0367
IONMODE: positive
RETENTIONTIME: 526.026
Links:
Comment:
Num Peaks: 8
85.0256 196
91.0503 2509
119.0454 2981
123.0441 1145
136.0722 15907
147.0421 383
165.0539 225
216.0298 1549
```

2. Upload and analysis in MASST.

Users can upload this file to MASST (https://gnps.ucsd.edu/ProteoSAFe/static/gnps-splash.jsp?redirect=auth) to perform repository mining. The users need to login first. Then, click the "**query spectrum**" button in MASST panel to start the analysis. Copy **related texts from MSP** file to "title", "precursor m/z", "spectrum input" panel in the web server, respectively.

Workflow Selection

Search Protocol: None | Reset Form | Save as Protocol

Title: M262T526

Workflow Description

SEARCH_SINGLE_SPECTRUM

Use MASST to query a single MS/MS spectrum across all public GNPS datasets. The mass spectrometry equivalent of NCBI BLAST helps to put the query spectrum in context of where else it occurs (including sample information) as well as search a single MS/MS spectrum against all public spectral libraries.

Workflow version release_29

Spectrum Input

Precursor M/Z: 262.0367

Spectrum Input:

```
85.0256 196
91.0503 2509
119.0454 2981
123.0441 1145
136.0722 15907
147.0421 383
165.0539 225
216.0298 1549
```

Modify the search parameters and click “submit” button. The **used parameters** in KGMN manuscript have been provided below.

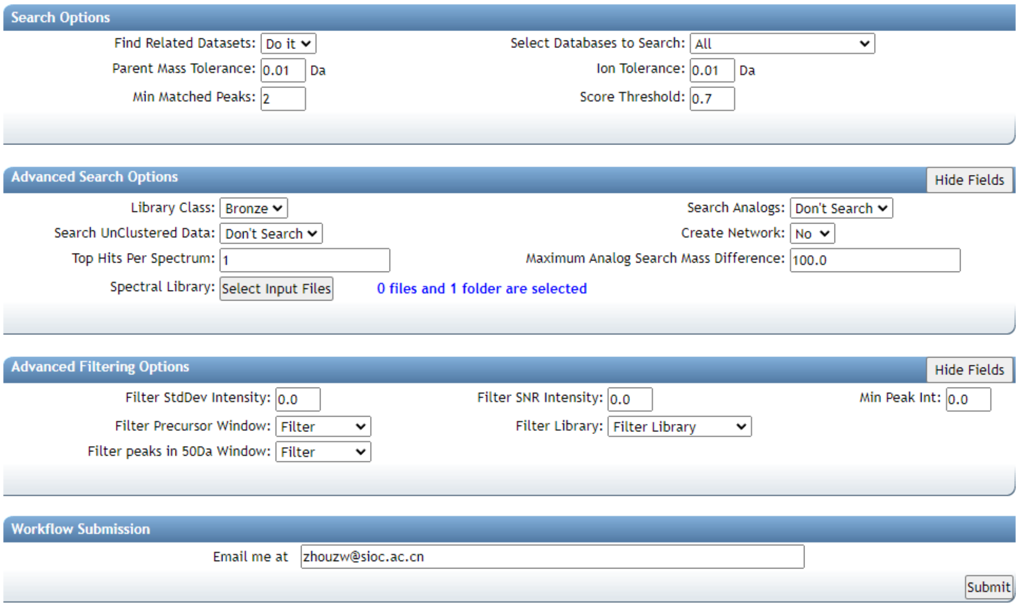

When the job finished, you will receive an email with a link. You can view and download results in the webserver.

- Matched data set: Dataset Matches → View File Matches → Download

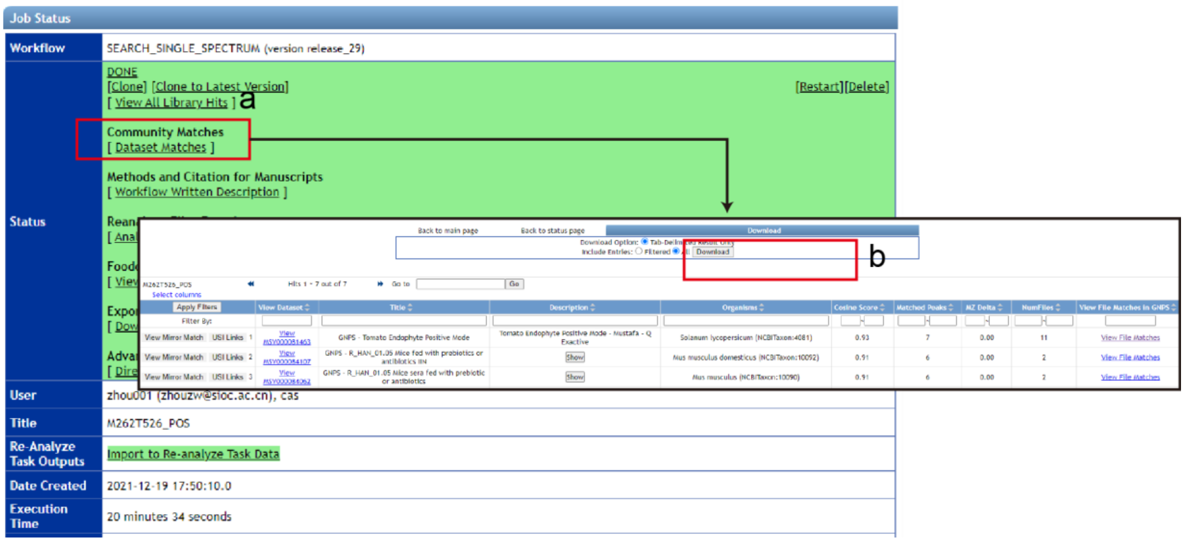

- Matched files: Dataset Matches → View File Matches → Download

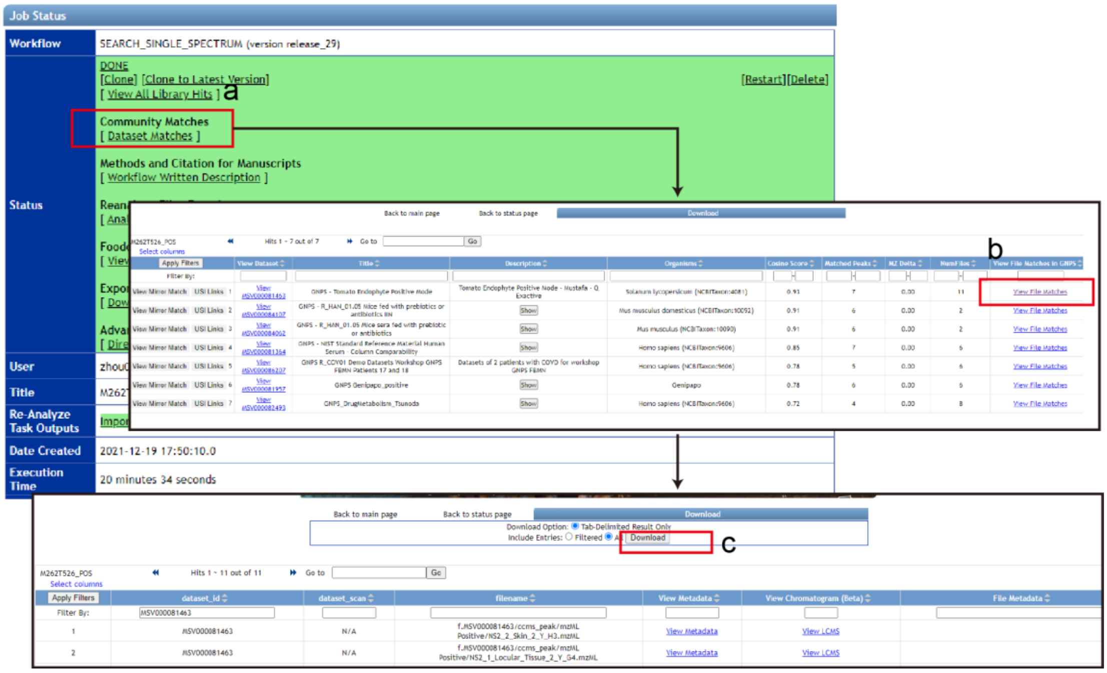

3. Result interpretation and visualization.

The downloaded results include 2 ZIP files, “view_all_datasets_matched.zip” and “view_all_file_datasets_matched.zip”. The files in packages can be further opened with Microsoft Office Excel or other program tools (e.g. R, Python).

- The table of “view_all_datasets_matched” contains meta information of appeared data sets, like “dataset description”, “dataset id”, “dataset organisms” and “files count”.

  Furthermore, we can conclude the species and sample information based on the dataset description. For our examples, it was appeared in 7 datasets, and 3 organisms (where genipapo is from human urine actually according to the data set description).

	A	B	C	D	E	F	G	H	I	J	K	L
1	dataset_description	dataset_filename	dataset_id	dataset_organisms	dataset_sc	dataset_title	files_count	matchedp	mzerror	score	specs_filer	specs_scan
2	Tomato Endophyte Po	continuous/clustered_data/	MSV000081463	Solanum lycopersicum (NCBIT	5708	GNPS - Tom	11	7	0.00129	0.926225	specs_ms.	1
3	Mice were fed with pre	continuous/clustered_data/	MSV000084107	Mus musculus domesticus (N(	11528	GNPS - R_H	2	6	0.00129	0.914833	specs_ms.	1
4	Control diet for C57Bl/	continuous/clustered_data/	MSV000084062	Mus musculus (NCBITaxon:10	11496	GNPS - R_H	2	6	0.00129	0.914833	specs_ms.	1
5	NIST SRM-1950 was p	continuous/clustered_data/	MSV000081364	Homo sapiens (NCBITaxon:96	7340	GNPS - NIS	6	7	0.00129	0.847013	specs_ms.	1
6	Datasets of 2 patients	continuous/clustered_data/	MSV000086207	Homo sapiens (NCBITaxon:96	435	GNPS R_CO	6	5	0.0017	0.780397	specs_ms.	1
7	notworking urines of c	continuous/clustered_data/	MSV000081957	Genipapo	765	GNPS Genip	6	6	0.00129	0.77843	specs_ms.	1
8	pilot data from a drug	continuous/clustered_data/	MSV000082493	Homo sapiens (NCBITaxon:96	87207	GNPS_DrugI	8	4	0.00129	0.715487	specs_ms.	1

- The table of “view_all_file_datasets_matched” contains names of matched files. Each file can be viewed online through the filename in GNPS dashboard (https://gnps-lcms.ucsd.edu/), while the files and dataset can be accessed in GNPS datasets (https://gnps.ucsd.edu/ProteoSAFe/datasets.jsp).

	A	B	C	D	E
1	basefilename	cluster_sca	dataset_id	filename	metadata
2	018c.mzML	435	MSV000086207	f.MSV000086207/ccms_peak/018c.mzML	
3	018b.mzML	435	MSV000086207	f.MSV000086207/ccms_peak/018b.mzML	
4	018a.mzML	435	MSV000086207	f.MSV000086207/ccms_peak/018a.mzML	
5	017c.mzML	435	MSV000086207	f.MSV000086207/ccms_peak/017c.mzML	
6	017b.mzML	435	MSV000086207	f.MSV000086207/ccms_peak/017b.mzML	
7	017a.mzML	435	MSV000086207	f.MSV000086207/ccms_peak/017a.mzML	
8	E12_3.mzML	11528	MSV000084107	f.MSV000084107/ccms_peak/E12_3.mzML	
9	E12_2.mzML	11528	MSV000084107	f.MSV000084107/ccms_peak/E12_2.mzML	
10	E12_3.mzML	11496	MSV000084062	f.MSV000084062/ccms_peak/E12_3.mzML	
11	E12_2.mzML	11496	MSV000084062	f.MSV000084062/ccms_peak/E12_2.mzML	
12	DM000088099_RB7_01_29	87234	MSV000082493	f.MSV000082493/ccms_peak/urine/DM000088099_RB	
13	DM000086580_RF12_01_2	87207	MSV000082493	f.MSV000082493/ccms_peak/urine/DM000086580_RF1	
14	DM000078719_RA11_01_2	87214	MSV000082493	f.MSV000082493/ccms_peak/urine/DM000078719_RA	
15	DM000078708_RC10_01_2	87214	MSV000082493	f.MSV000082493/ccms_peak/urine/DM000078708_RC	
16	DM000078265_RD7_01_2	87207	MSV000082493	f.MSV000082493/ccms_peak/urine/DM000078265_RD	
17	DM000076834_RB8_01_29	87230	MSV000082493	f.MSV000082493/ccms_peak/urine/DM000076834_RB	
18	DM000076821_RC12_01_2	87234	MSV000082493	f.MSV000082493/ccms_peak/urine/DM000076821_RC	
19	DM000076799_RC8_01_2	87230	MSV000082493	f.MSV000082493/ccms_peak/urine/DM000076799_RC	
20	Urine83_Juice_12h_Top3_	765	MSV000081957	f.MSV000081957/ccms_peak/Urine83_Juice_12h_Top3_	

With above information, it would be easy to reproduce figures of repository validation. The result of above example can be downloaded **here** (https://mega.nz/file/R6oCiITS#L8uZQnjb4wx65IuVnWvcCKXL8ZIPLM36ExyvXR7aY3E).

Supplementary Note 3.

Tutorial of integrating KGMN results with other in-silico MS/MS workflows

Zhiwei Zhou

2022-06-10

Introduction

Knowledge-guided multi-layer network (KGMN) is a new approach leveraging knowledge-guided multi-layer networks to annotate known and unknown metabolites in untargeted metabolomics data. Although KGMN is an independent software tool, it can further integrate with other workflows to help users discover and validate metabolites. This tutorial aims to provide an easy instruction to integrated KGMN results with 3 common in-silico MS/MS tools (MetFrag, CFM-ID, MS-FINDER).

Here, we mainly focus on providing ways to help users linking KGMN with other tools. It should be note that the parameters need to be adjusted according to their instrument settings and experimental designs. **The detailed usage please refer their own tutorials.**

Tutorials:

- MetFrag: https://ipb-halle.github.io/MetFrag/
- CFM-ID: https://cfmid.wishartlab.com/
- MSFINDER: https://mtbinfo-team.github.io/mtbinfo.github.io/MS-FINDER/tutorial.html

Demo datasets:

- NIST urine set (Positive mode, processed by KGMN): **Download** (https://mega.nz/file/w7ZnjLAa#u4Dj5lhkYyEhOZHH4BX_HUHvGMkjZ_ti5bn986tgyrY)

If you use these tools, please cite their papers (MetFrag[2], CFM-ID[3], MSFINDER[4]).

1. Installation.

This integration of KGMN and in-silico MS/MS tools is mainly performed by R package “MetDNA2InSilicoTool”. It can be downloaded as below:

```
# Install required packages
if(!require(devtools)){
install.packages("devtools")
}

if(!require(BiocManager)){
install.packages("BiocManager")
}

# Install CRAN/Bioconductor packages
required_pkgs <- c("dplyr","tidyr","readr","stringr","rcdk")
list_installed <- installed.packages()

new_pkgs <- required_pkgs[!(required_pkgs %in% list_installed[,'Package'])]
if (length(new_pkgs) > 0) {
  BiocManager::install(new_pkgs)
} else {
  cat('Required CRAN/Bioconductor packages installed\n')
}


# Install GitHub packages - call MetFrag
devtools::install_github("schymane/ReSOLUTION")

# Install GitHub packages
devtools::install_github("ZhuMetLab/MetDNA2InSilicoTool")
```

2. MetFrag

MetFrag is a common in-silico MS/MS tool developed by *Dr. Sebastian Wolf* and *Dr. Christoph Ruttkies*. It provides multiple ways to use it, including web server (MetFragWeb), MetFrag

commandline tool (MetFragCL) and R package (MetFragR). In this workflow, we mainly use **MetFragCL (version 2.4.5)** to demonstrate the connection between KGMN and MetFrag.

2.1 Download MetFragCL program.

MetFragCL is a Java Archive File. It can be downloaded from GitHub. https://github.com/ipb-halle/MetFragRelaunched/releases/tag/v2.4.8

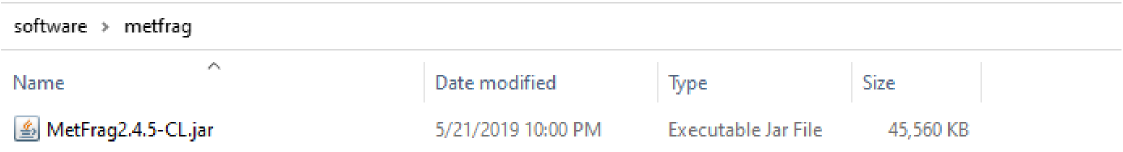

Note: The MetFragCL program is depended on **Java**. Please install java and set environment variable first.

2.2 Load required packages, and setting the working directory.

We use MetDNA2InSilicoTool to call MetFragCL. Please set the working directory at 07_insilico_msms, which is localized at KGMN result folder. Then, load some required packages.

```
# set working directory
setwd('G:/00_projects/03_MetDNA2/00_data/20220609_insilico_ms2_demo/NIST_urine_pos/07_insilico_msms/')

# load packages
library(dplyr)
library(MetDNA2InSilicoTool)

# reformat identification_table
reformatTable1(dir_path =
'G:/00_projects/03_MetDNA2/00_data/20220609_insilico_ms2_demo/NIST_urine_pos/07_insilico_msms/')
```

It looks like as below:

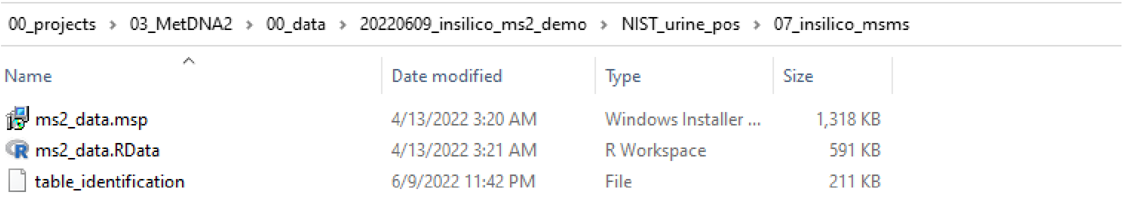

2.3 Generate input files for your interested peak.

In this workflow, users need generate necessary files for different in-silico tools. Here, we use an interesting peak **M196T420** as example (Figure 4c). This peak is annotated as an unknown peak in KGMN, while it has 6 possible metabolite candidates.

First, generate necessary file for M196T420.

```
# generate files for in-silico MS/MS match
# peak 'M196T420' as example
generateFiles4InsilicoMsMs(peak_id = 'M196T420',
                                        dir_path =
'G:/00_projects/03_MetDNA2/00_data/20220609_insilico_ms2_demo/NIST_urine_pos/07_insilico_
msms/')
```

A folder "M196T420" will be created as blow:

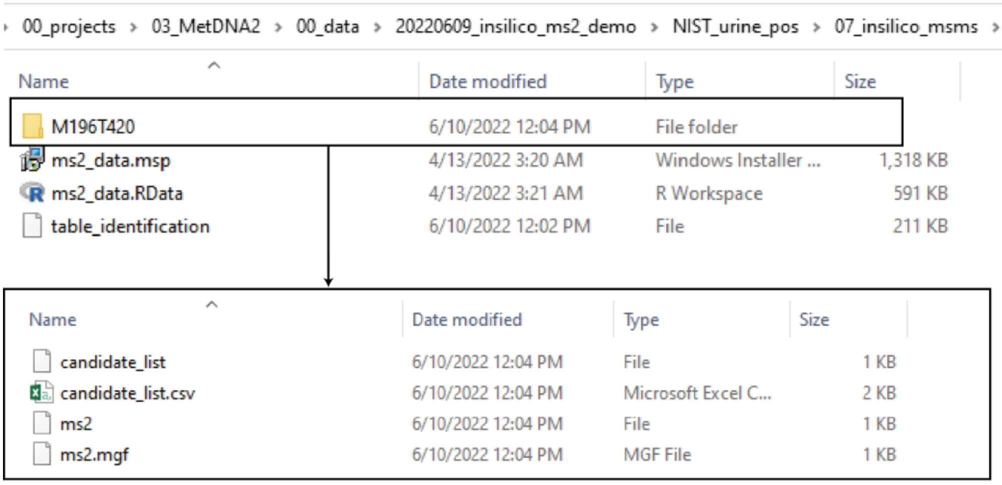

It contains two files, candidate_list and MS/MS file. The **candidate list** is a list of chemical structures for in-silico MS/MS tool validation. The **MS/MS file** is a experimental spectrum of the targeted peak. The MS/MS file can be used for other in-silico tools if needed.

2.4 Run MetFrag.

We provide a R function (runMetFragMatch) to call MetFragCL. Here, the path of MetFragCL should be given. Other parameters can be adjusted. In MetDNA2InSilicoTool package, we only open limited parameters. For advanced users, the parameters can be adjusted according to MetFragCL tutorial.

```
# run MetFrag

# parameters
# peak_id: name of interested peak
# metfrag_path: path of metfrag program
# ppm: relative error of precursor MS1. 25 ppm
# mzabs: absolute error or MS1. 0.01 Da
# frag_ppm: relative error of precursor MS1. 25 ppm

runMetFragMatch(peak_id = 'M196T420',
                dir_path =
'G:/00_projects/03_MetDNA2/00_data/20220609_insilico_ms2_demo/NIST_urine_pos/07_insilico_msms/',
                metfrag_path = 'F:/software/metfrag/MetFrag2.4.5-CL.jar',
                ppm = 25,
                mzabs = 0.01,
                frag_ppm = 25)
```

2.5 Output of MetFrag.

A folder “01_metfrag” is created in the “M196T420” folder. It contains results of MetFrag. For candidate with different adducts, they are divided into different folders. The rank results localize at the subfolder “results”.

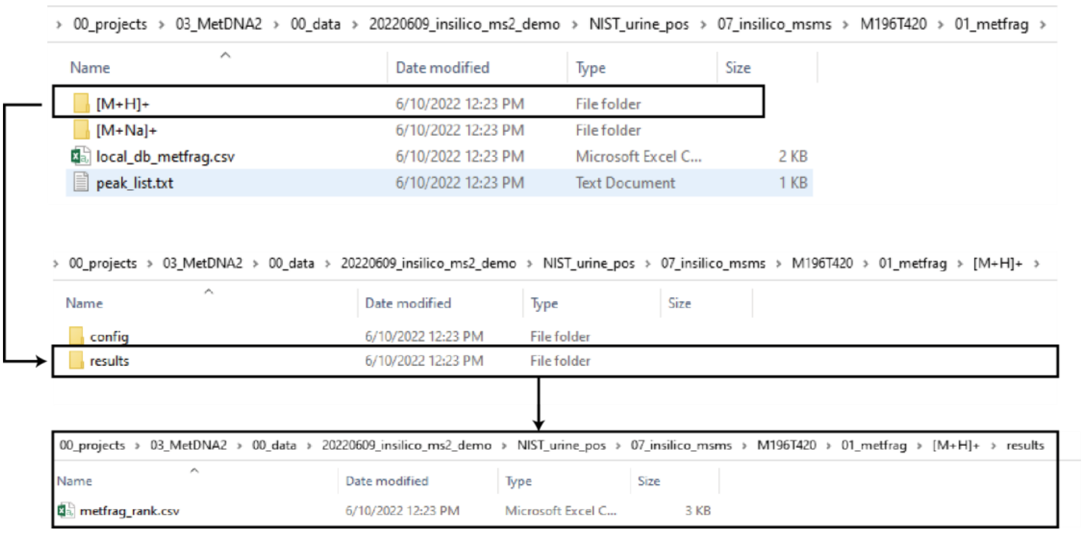

3. CFM-ID

CFM-ID is a machine-learning based MS/MS prediction tool, which developed by *Prof. David S Wishart Lab*. It provides several access ways, including web server and command lines. In this workflow, we mainly use CFM-ID (version 2.4) to demonstrate the connection between KGMN and CFM-ID

.

3.1 Download and Set CFM-ID program.

Here, we utilize CFM-ID (v2.4). The program can be downloaded at here (https://sourceforge.net/projects/cfm-id/files/). The new docker image of CFM-ID4 is available at here (https://bitbucket.org/wishartlab/cfm-id-code/src/master/).

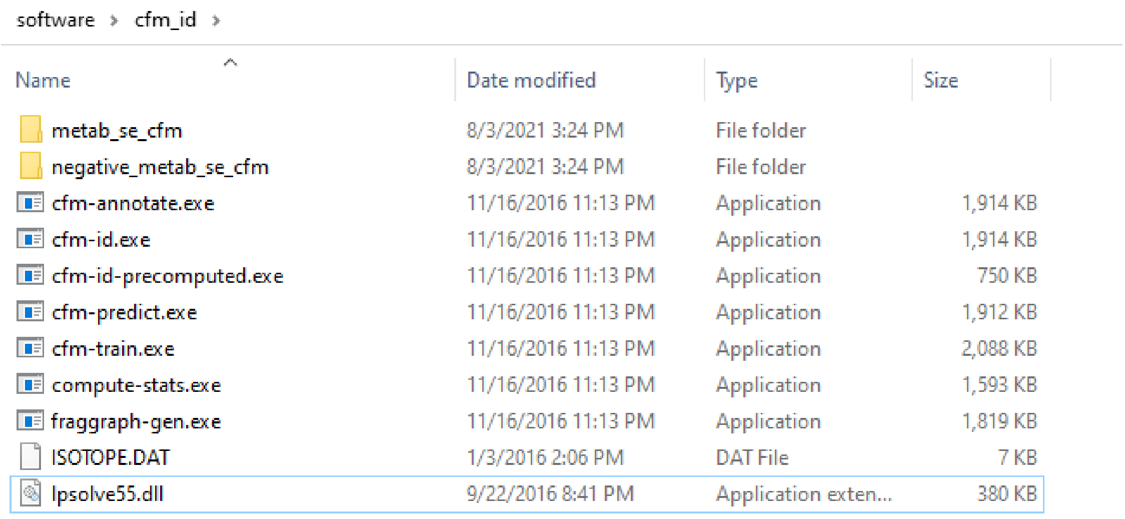

Note:

- The prediction model is required for CFM-ID. Users can train their own model or directly use the pre-trained model. The predicted model can be downloaded at **here** (https://sourceforge.net/p/cfm-id/code/HEAD/tree/supplementary_material/trained_models/esi_msms_models/).

3.2 Load required packages, and setting the working directory.

Similar with MetFrag, we use MetDNA2InSilicoTool to call CFM-ID. Please set the working directory at 07_insilico_msms, which is localized at KGMN result folder. Then, load some required packages.

```
# set working directory
setwd('G:/00_projects/03_MetDNA2/00_data/20220609_insilico_ms2_demo/NIST_urine_pos/07_insilico_msms/')

# load packages
library(dplyr)
library(MetDNA2InSilicoTool)

# reformat identification_table
reformatTable1(dir_path =
'G:/00_projects/03_MetDNA2/00_data/20220609_insilico_ms2_demo/NIST_urine_pos/07_insilico_msms/')
```

3.2 Generate input files for your interested peak.

This step is consistent with MetFrag. We use an interesting peak M196T420 as example.

```
# generate files for in-silico MS/MS match
# peak 'M196T420' as example
generateFiles4InsilicoMsMs(peak_id = 'M196T420',
                           dir_path =
'G:/00_projects/03_MetDNA2/00_data/20220609_insilico_ms2_demo/NIST_urine_pos/07_insilico_msms/')
```

3.3 Run CFM-ID.

```
# run CFM-ID

# parameters
# cfmid_path: path of cfm-id
# config_file: config file of prediction model. It should be selected according to ionzation polairty. Pos: metab_se_cfm/param_config.txt; Neg: negative_metab_se_cfm/param_config.txt
# param_file: parameter file of prediction model. It should be selected according to ionzation polairty. Pos: metab_se_cfm/param_output0.log; Neg: negative_metab_se_cfm/param_output0.log
# score_type: rank score of CFM-ID. Default: 'jaccard'
# ppm: relative mz tolerance
# mzabs: absolute mz tolerance

runCfmIdMatch(peak_id = 'M196T420',
              dir_path = 
'G:/00_projects/03_MetDNA2/00_data/20220609_insilico_ms2_demo/NIST_urine_pos/07_insilico_msms/',
              cfmid_path = 'F:/software/cfm_id/cfm-id.exe',
              config_file = 'F:/software/cfm_id/metab_se_cfm/param_config.txt',
              param_file = 'F:/software/cfm_id/metab_se_cfm/param_output0.log',
              score_type = 'Jaccard',
              ppm = 25,
              mzabs = 0.01)
```

3.4 Output of CFM-ID.

A folder “02_cfmid” will be created in the “M196T420” folder. It contains results of CFM-ID. The “cfmid_result.txt” is the CFM-ID rank result. The “cfmid_pred_spec.msp” is the predicted MS/MS spectra of candidates.

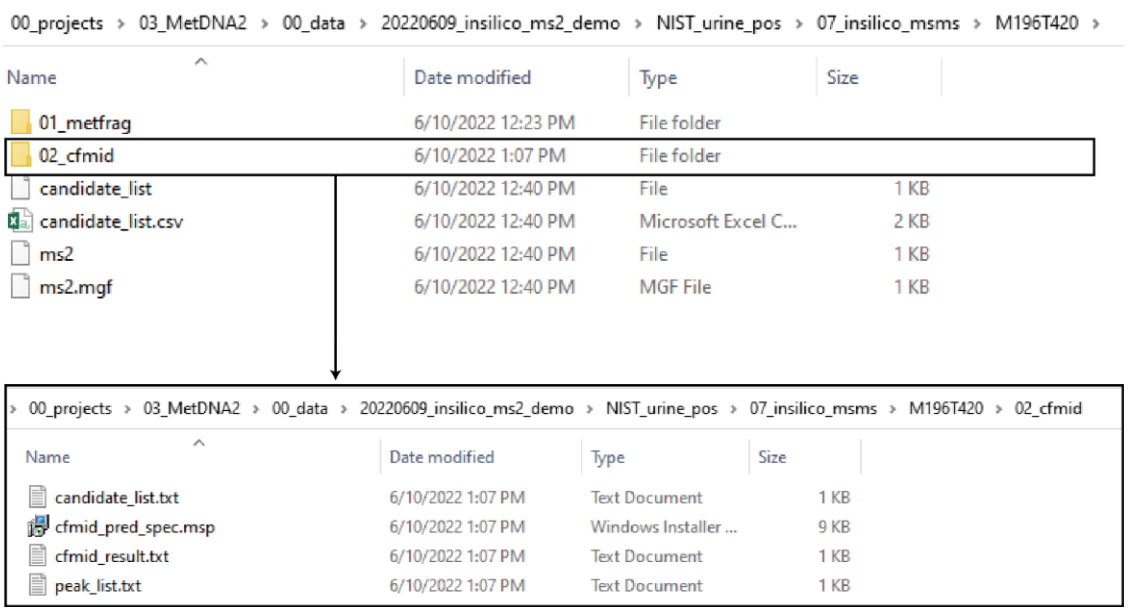

4. MS-FINDER

MS-FINDER is a rule-based fragmentation tool, which developed by *Prof. Hiroshi Tsugawa* and *Prof. Masanori Arita* Lab. It usually is combined with MS-DIAL. In this tutorial, we mainly used it command tool (version 3.2.4) to evaluate KGMN metabolites.

4.1 Download MS-FINDER program.

We used the MS-FINDER v3.24. The newest version can be downloaded from here.

Note: The instruction of MetDNA2InSilicoTool is only supported and tested in Windows System.

software > MSFINDER > MSFINDER_ver_3.24

Name	Date modified	Type	Size
IKVM.OpenJDK.Text.dll	1/15/2015 3:02 PM	Application exten...	801 KB
IKVM.OpenJDK.Util.dll	1/15/2015 3:02 PM	Application exten...	1,950 KB
IKVM.OpenJDK.XML.API.dll	1/15/2015 3:02 PM	Application exten...	201 KB
IKVM.OpenJDK.XML.Parse.dll	1/15/2015 3:02 PM	Application exten...	2,619 KB
IKVM.Runtime.dll	1/15/2015 3:02 PM	Application exten...	1,016 KB
IKVM.Runtime.JNI.dll	1/15/2015 3:02 PM	Application exten...	76 KB
IsotopeRatioCalculator.dll	6/2/2019 5:13 PM	Application exten...	32 KB
MassLynxRaw.dll	5/10/2018 10:39 AM	Application exten...	738 KB
MassLynxRawSDK.dll	5/10/2018 10:39 AM	Application exten...	24 KB
MassSpectrogram.dll	6/10/2019 5:04 PM	Application exten...	97 KB
MassSpectrogram.dll.config	9/20/2018 11:43 AM	CONFIG File	4 KB
Mathematics.dll	5/5/2016 12:04 PM	Application exten...	24 KB
MessagePack.dll	1/30/2018 3:19 PM	Application exten...	273 KB
MolecularFormulaFinder.dll	6/10/2019 5:02 PM	Application exten...	135 KB
MsdialGcmsProcess.dll	6/10/2019 5:03 PM	Application exten...	156 KB
MsdialLcmsProcess.dll	6/10/2019 5:03 PM	Application exten...	324 KB
MSFINDER.exe	6/10/2019 5:04 PM	Application	1,235 KB
MSFINDER.exe.config	9/20/2018 11:43 AM	CONFIG File	4 KB
MSFINDER.INI	5/28/2019 11:57 AM	Configuration sett...	3 KB
MsfinderCommon.dll	6/10/2019 5:04 PM	Application exten...	54 KB
MsfinderConsoleApp.exe	6/10/2019 5:02 PM	Application	194 KB
MsfinderConsoleApp.exe.config	11/21/2018 5:36 PM	CONFIG File	4 KB

4.2 Load required packages, and setting the working directory.

Repeat procedures in MetFrag and CFIM-ID. Set the working directory at 07_insilico_msms, which is localized at KGMN result folder. Then, load some required packages.

```
# set working directory
setwd('G:/00_projects/03_MetDNA2/00_data/20220609_insilico_ms2_demo/NIST_urine_pos/07_insilico_msms/')

# load packages
library(dplyr)
library(MetDNA2InSilicoTool)

# reformat identification_table
reformatTable1(dir_path =
'G:/00_projects/03_MetDNA2/00_data/20220609_insilico_ms2_demo/NIST_urine_pos/07_insilico_msms/')
```

4.3 Generate input files for your interested peak.

Consist with **MetFrag** and **CFM-ID**, generate related files for targeted peaks. The example M196T420 is here.

```
# generate files for in-silico MS/MS match
# peak 'M196T420' as example
generateFiles4InsilicoMsMs(peak_id = 'M196T420',
                           dir_path = 
'G:/00_projects/03_MetDNA2/00_data/20220609_insilico_ms2_demo/NIST_urine_pos/07_insilico_
msms/')
```

4.4 Run MS-FINDER

We provided a R function (runMsFinderMatch) to call MS-FINDER. Here, we use the command tool of MS-FINDER (MsfinderConsoleApp.exe). The path of MS-FINDER should be given.

```
# run MS-FINDER

# parameters
#
runMsFinderMatch(peak_id = 'M196T420',
                 dir_path = 
'G:/00_projects/03_MetDNA2/00_data/20220609_insilico_ms2_demo/NIST_urine_pos/07_insilico_
msms',
                 msfinder_path = 
'F:/software/MSFINDER/MSFINDER_ver_3.24/MsfinderConsoleApp.exe')
```

4.5 Output of MS-FINDER.

A folder “03_msfinder” will be created in the “M196T420” folder. It contains results of MS-FINDER. The result of MS-FINDER is organized as adduct types. The rank result will be 03_msfinder -> [M+H]+ -> result -> Structure result-2055.txt.

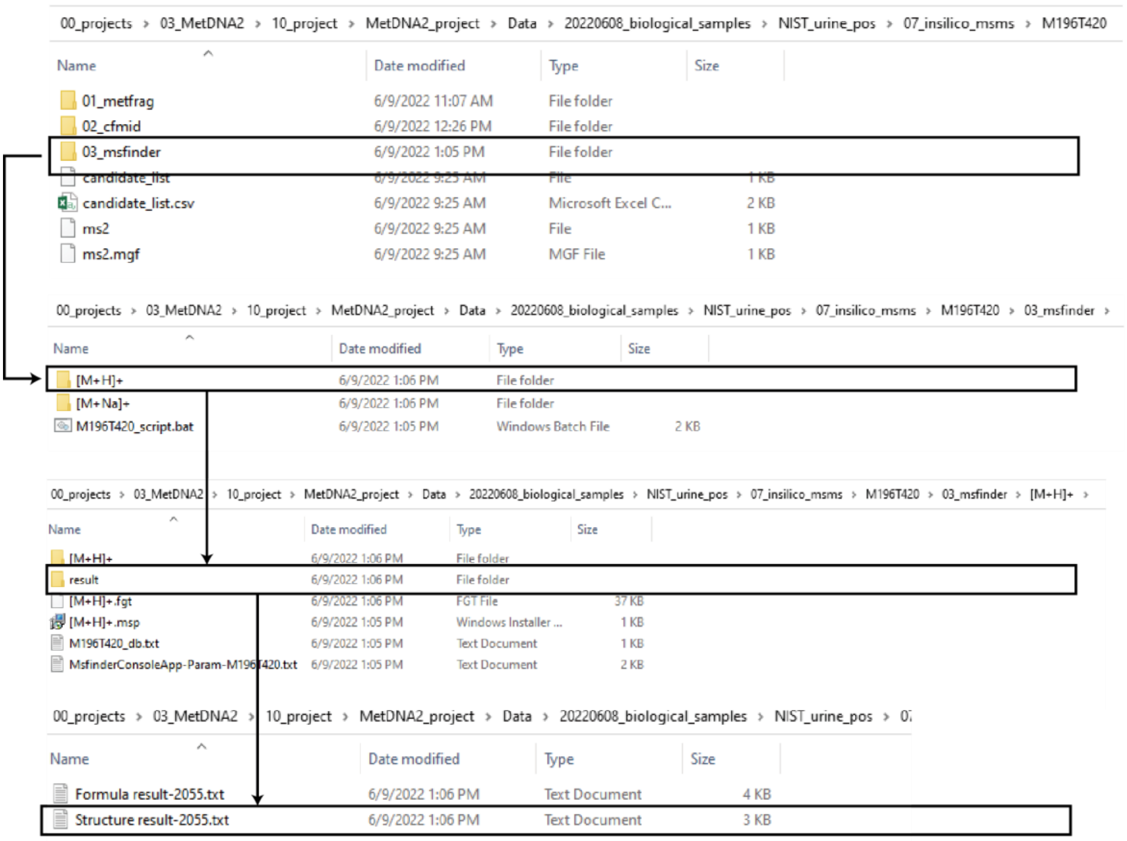

Note:

- The parameter file of MS-FINDER is in '/03_msfinder/[M+H]+/MsfinderConsoleApp-param.txt'. Advanced users can adjust this file, and rerun MS-FINDER.

5. The script for connection KGMN and in-silico MS/MS tools

Here is a script contains above codes to help to connect KGMN and in-silico MS/MS tools quickly.

```
# set working directory
setwd('G:/00_projects/03_MetDNA2/00_data/20220609_insilico_ms2_demo/NIST_urine_pos/')

# load packages
library(dplyr)
library(MetDNA2InSilicoTool)

# copy files
copyFiles4InsilicoTool(dir_path = '.')

# set working directory
setwd('G:/00_projects/03_MetDNA2/00_data/20220609_insilico_ms2_demo/NIST_urine_pos/07_in
silico_msms/')

# reformat identification_table
reformatTable1(dir_path = '.')


# generate files for in-silico MS/MS match
# peak 'M196T420' as example
generateFiles4InsilicoMsMs(peak_id = 'M196T420')

# run MetFrag
runMetFragMatch(peak_id = 'M196T420',
                metfrag_path = 'F:/software/metfrag/MetFrag2.4.5-CL.jar',
                ppm = 25,
                mzabs = 0.01,
                frag_ppm = 25)


# run CFM-ID
runCfmIdMatch(peak_id = 'M196T420',
              cfmid_path = 'F:/software/cfm_id/cfm-id.exe',
```

```
                config_file = 'F:/software/cfm_id/metab_se_cfm/param_config.txt',
                param_file = 'F:/software/cfm_id/metab_se_cfm/param_output0.log',
                score_type = 'Jaccard',
                ppm = 25,
                mzabs = 0.01)

# run MS-FINDER
# note: the dir_path must be given
runMsFinderMatch(peak_id = 'M196T420',
                 dir_path =
'G:/00_projects/03_MetDNA2/00_data/20220609_insilico_ms2_demo/NIST_urine_pos/07_insilico_
msms',
                 msfinder_path =
'F:/software/MSFINDER/MSFINDER_ver_3.24/MsfinderConsoleApp.exe')
```

Supplementary References:

1. Wang, M. et al. Mass spectrometry searches using MASST. *Nat. Biotechnol.* **38**, 23–26 (2020).
2. Ruttkies, C., Schymanski, E. L., Wolf, S., Hollender, J. & Neumann, S. MetFrag relaunched: incorporating strategies beyond in silico fragmentation. *J. Cheminform.* **8**, 3 (2016).
3. Wang, F. et al. CFM-ID 4.0: More Accurate ESI-MS/MS Spectral Prediction and Compound Identification. *Anal. Chem.* **93**, 11692-11700 (2021).
4. Tsugawa, H. et al. Hydrogen Rearrangement Rules: Computational MS/MS Fragmentation and Structure Elucidation Using MS-FINDER Software. *Anal. Chem.* **88**, 7946–7958 (2016).
