# Supplementary material for: Metabolite annotation from knowns to unknowns through knowledge-guided multi-layer metabolic networking
Source: Nat Commun. 2022 Nov 4;13:6656. doi: 10.1038/s41467-022-34537-6 (PMC9636193; doi:10.1038/s41467-022-34537-6)
Supplement: Supplementary file 3 — Description of Additional Supplementary Files [file 41467_2022_34537_MOESM3_ESM.docx]

File Name: Supplementary Data 1

Description: Peak annotation evaluation between MetDNA1 and KGMN (MetDNA2).

File Name: Supplementary Data 2

Description: 46 standard mixture (46std_mix) and the knowledge-based metabolic reaction network.

File Name: Supplementary Data 3

Description: KGMN results of 46std_mix data set and validation results.

File Name: Supplementary Data 4

Description: KGMN results of NIST urine data sets and validation results.

File Name: Supplementary Data 5

Description: KGMN results of different biological samples.

File Name: Supplementary Data 6

Description: Recurrent unknowns of NIST urine via repository-mining.

File Name: Supplementary Data 7

Description: Table of adducts, neutral losses, empirical rules in KGMN.

File Name: Supplementary Data 8

Description: Knowledge-based metabolic reaction network.

File Name: Supplementary Data 9

Description: Network files of supplementary figures.
