# Supplementary material for: Metabolite annotation from knowns to unknowns through knowledge-guided multi-layer metabolic networking
Source: Nat Commun. 2022 Nov 4;13:6656. doi: 10.1038/s41467-022-34537-6 (PMC9636193; doi:10.1038/s41467-022-34537-6)
Supplement: Supplementary file 13 — Reporting Summary [file 41467_2022_34537_MOESM13_ESM.pdf]

nature portfolio

Corresponding author(s): Zheng-Jiang Zhu

Last updated by author(s): Oct 21, 2022

Reporting Summary

Nature Portfolio wishes to improve the reproducibility of the work that we publish. This form provides structure for consistency and transparency in reporting. For further information on Nature Portfolio policies, see our Editorial Policies and the Editorial Policy Checklist.

Statistics

For all statistical analyses, confirm that the following items are present in the figure legend, table legend, main text, or Methods section.

n/a	Confirmed	
☐	☒	The exact sample size (*n*) for each experimental group/condition, given as a discrete number and unit of measurement
☒	☐	A statement on whether measurements were taken from distinct samples or whether the same sample was measured repeatedly
☒	☐	The statistical test(s) used AND whether they are one- or two-sided<br>*Only common tests should be described solely by name; describe more complex techniques in the Methods section.*
☒	☐	A description of all covariates tested
☒	☐	A description of any assumptions or corrections, such as tests of normality and adjustment for multiple comparisons
☐	☒	A full description of the statistical parameters including central tendency (e.g. means) or other basic estimates (e.g. regression coefficient) AND variation (e.g. standard deviation) or associated estimates of uncertainty (e.g. confidence intervals)
☒	☐	For null hypothesis testing, the test statistic (e.g. *F*, *t*, *r*) with confidence intervals, effect sizes, degrees of freedom and *P* value noted<br>*Give P values as exact values whenever suitable.*
☒	☐	For Bayesian analysis, information on the choice of priors and Markov chain Monte Carlo settings
☒	☐	For hierarchical and complex designs, identification of the appropriate level for tests and full reporting of outcomes
☐	☒	Estimates of effect sizes (e.g. Cohen's *d*, Pearson's *r*), indicating how they were calculated

Our web collection on statistics for biologists contains articles on many of the points above.

Software and code

Policy information about availability of computer code

Data collection	Analyst® TF 1.6 Software was used for LC-MS/MS data acquisition
Data analysis	R (4.0.3), BioTransformer (version 1.0.8), ProteoWizard (version 3.0.6150), XCMS (version 1.46.0), MetDNA (version 1.3.2), MetFrag (version 2.4.5-CL), CFM-ID (version 2.4), MS-FINDER (version 3.24), MASST (release_29), R package CAMERA (version 1.46.0), R package caret (version 6.0.90), Cytoscape (version 3.8), R package rcdk (version 3.4.7.1), MestReNova (v9.0.1), MetDNA2 (v1.0.7, https://github.com/ZhuMetLab/MetDNA2), MetDNA2InSilicoTools (v0.1.1, https://github.com/ZhuMetLab/MetDNA2InSilicoTool), MetDNA2Vis(v0.1.1, https://github.com/ZhuMetLab/MetDNA2Vis)

For manuscripts utilizing custom algorithms or software that are central to the research but not yet described in published literature, software must be made available to editors and reviewers. We strongly encourage code deposition in a community repository (e.g. GitHub). See the Nature Portfolio guidelines for submitting code & software for further information.

Data

Policy information about availability of data

All manuscripts must include a data availability statement. This statement should provide the following information, where applicable:

- Accession codes, unique identifiers, or web links for publicly available datasets
- A description of any restrictions on data availability
- For clinical datasets or third party data, please ensure that the statement adheres to our policy

The databases were used in this study: KEGG reaction pair database (downloaded at 7th March, 2017), Human Metabolome Database (v4.0, released on 18th

December, 2018).

All the metabolomics datasets described in our study can be downloaded from the MetDNA2 website [http://metdna.zhulab.cn/]. The raw data files of NIST human urine, NIST human plasma, and BV2 cell can be accessed at the National Omics Data Encyclopedia under Accession Code OEP003157 [https://www.biosino.org/node/project/detail/OEP003157]. The raw data of in-vitro metabolism can be accessed at National Omics Data Encyclopedia under Accession Code OEP003284 [https://www.biosino.org/node/project/detail/OEP003284]. The raw data of fruit fly head are available at MetaboLights under Accession Code MTBLS612 [https://www.ebi.ac.uk/metabolights/MTBLS612] and MTBLS615 [https://www.ebi.ac.uk/metabolights/MTBLS615]. The raw data of mouse liver are available at MetaboLights under Accession Code MTBLS601 [https://www.ebi.ac.uk/metabolights/MTBLS601] and MTBLS606 [https://www.ebi.ac.uk/metabolights/MTBLS606]. The supplementary data files can be accessed at Zenodo (DOI: 10.5281/zenodo.6784047). Source data are provided with this paper.

Human research participants

Policy information about studies involving human research participants and Sex and Gender in Research.

Reporting on sex and gender	No human participant is involved in this study.
Population characteristics	No human participant is involved in this study.
Recruitment	No human participant is involved in this study.
Ethics oversight	No human participant is involved in this study.

Note that full information on the approval of the study protocol must also be provided in the manuscript.

Field-specific reporting

Please select the one below that is the best fit for your research. If you are not sure, read the appropriate sections before making your selection.

☒ Life sciences ☐ Behavioural & social sciences ☐ Ecological, evolutionary & environmental sciences

For a reference copy of the document with all sections, see nature.com/documents/nr-reporting-summary-flat.pdf

Life sciences study design

All studies must disclose on these points even when the disclosure is negative.

Sample size	One biological sample was used for the standard reference samples (i.e. NIST human urine, NIST human plasma). The experiments of BV2 cell were performed with 8 independent samples for each group. No sample-size calculation was performed here, and 8 independent biological replicates represent enough biological variations in most biological studies.
Data exclusions	No samples were excluded from analysis in this study.
Replication	The standard reference samples (i.e. NIST human urine, NIST human plasma) were repeated with 5 technical replications (injections). The BV2 cell samples (n=8, biologically independent samples) were used in analysis. All attempts at replication were successful.
Randomization	For standard reference samples, the randomization is not required, because only one standard sample was used. For BV2 cell data set, samples were assigned randomly to acquire LC-MS data.
Blinding	It did not require the use of blinding, because these datasets were used to demonstrate software applicability instead of generating biological results.

Reporting for specific materials, systems and methods

We require information from authors about some types of materials, experimental systems and methods used in many studies. Here, indicate whether each material, system or method listed is relevant to your study. If you are not sure if a list item applies to your research, read the appropriate section before selecting a response.

Materials & experimental systems

n/a	Involved in the study
☒	☐ Antibodies
☐	☒ Eukaryotic cell lines
☒	☐ Palaeontology and archaeology
☒	☐ Animals and other organisms
☒	☐ Clinical data
☒	☐ Dual use research of concern

Methods

n/a	Involved in the study
☒	☐ ChIP-seq
☒	☐ Flow cytometry
☒	☐ MRI-based neuroimaging

Eukaryotic cell lines

Policy information about cell lines and Sex and Gender in Research

Cell line source(s)	BV2 cell lines were originally purchased from ATCC with product number CRL-3265.
Authentication	Authentication was done using STR (Short Tandem Repeat) analysis.
Mycoplasma contamination	The cell lines were not tested for mycoplasma contamination.
Commonly misidentified lines (See ICLAC register)	No commonly misidentified cells were used.
